# Traversing the canopy: phenology-driven changes and within-canopy transport shape the phyllosphere microbiome in a temperate floodplain hardwood forest

**DOI:** 10.1186/s40793-026-00984-7

**Published:** 2026-11-12

**Authors:** Dinesh Sanka Loganathachetti, Beate Michalzik, Marina M. Sandoval, Patrick Zerhusen, Ronny Richter, Rolf A. Engelmann, Tom Künne, Christian Wirth, Kirsten Küsel, Martina Herrmann

**Affiliations:** 1 Institute of Biodiversity, Ecology and Evolution, Aquatic Geomicrobiology, Friedrich Schiller University Jena, Rosalind-Franklin-Str. 5, 07745 Jena, Germany; 2 German Center for Integrative Biodiversity Research Halle-Jena-Leipzig - iDiv, Puschstr. 4, 04103 Leipzig, Germany; 3 Institute of Geography, Soil Science, Friedrich Schiller University Jena, Löbdergraben 32, 07743 Jena, Germany; 4 Institute for Biology, Systematic Botany and Functional Biodiversity, Leipzig University, Johannisallee 21, 04103 Leipzig, Germany; 5 Institute for Geography, Geoinformatics and Remote Sensing, Leipzig University, Johannisallee 19a, 04013 Leipzig, Germany; 6 Cluster of Excellence “Balance of the Microverse”, Friedrich Schiller University Jena, Rosalind-Franklin-Str. 5, 07745 Jena, Germany

**Keywords:** Phyllosphere, Throughfall, Temperate forest, Drift and dispersal limitation

## Abstract

Phyllosphere microbiomes are subject to microbial import from various sources and undergo substantial changes during phenological changes of plants. However, especially for forest canopies, these processes are still poorly understood. We disentangled the controls of phenology-driven changes versus tree species identity on the phyllosphere microbiome in a temperate floodplain forest and assessed the role of transport processes in shaping distribution patterns within the canopy. Leveraging the infrastructure of the Leipzig canopy crane (Central Germany), we followed bacterial community composition and rainwater-mediated bacterial transport for oak (*Quercus robur* L.), ash (*Fraxinus excelsior* L.) and linden (*Tilia cordata* MILL) across early (May), mid (July) and late (October) phenological stages. These stages explained 23% of phyllosphere bacterial community variation, followed by tree species identity (13%) and canopy position (2%). Phyllosphere bacterial community assembly was mostly driven by dispersal limitation at the early phenological stage while communities became more homogeneous and functionally redundant during mid and late phenological stages, along with a strong decline of plant pathogens and increasing potential for microbially mediated biocontrol mechanisms. Throughfall-mediated transport of bacteria increased substantially from March to May, indicating the strong influence of wash-off effects from the emerging foliage. Our findings demonstrate that in temperate forests, phenology-driven effects on the phyllosphere microbiome exceed tree species’ specific effects. Extent and selectivity of throughfall-mediated mobilization of microbes adds another layer of complexity to the spatial heterogeneity of microbial communities in tree crowns.

**Supplementary Information:**

The online version contains supplementary material available at https://doi.org/10.1186/s40793-026-00984-7.

## Introduction

Tree canopies represent the interface to the atmosphere and are exposed to the input of energy, water and matter, spanning a surface area of approximately 4 × 10^8^ km^2^ on a global scale [1]. Trees harbour habitats on leaves (phyllosphere) and bark, which host microbial communities involved in central biogeochemical processes as well as plant-microbe interactions, affecting plant community dynamics, ecosystem functioning and productivity [2]. Bacteria present in the phyllosphere interact with each other, the plant host, and the environment through competition, commensalism, antagonism and neutral interactions [3]. Interactions with the host include various functions beneficial to plants such as nitrogen fixation [4], methane oxidation [5], and phosphate solubilization [6]. In turn, pathogenic interactions may decrease the host’s fitness [7].

Tree species identity, canopy position, and phenology have been reported as drivers for phyllosphere bacterial communities in temperate forests [8–11]. However, the relative importance of these controls is still poorly understood. Technical challenges in accessing the whole tree crown for sampling have so far largely constrained our understanding of vertical patterns of the phyllosphere microbiome [11, 12]. Especially the canopy top represents a key interface between the forest and the surrounding atmospheric environments, mediating microbial and chemical exchange while its phyllosphere microbiome is the most exposed to harsh weather conditions and UV radiation.

Host physiology and leaf chemistry are central factors underlying the influence of tree species identity on phyllosphere bacterial communities. These host-specific properties are subject to phenology-driven changes, e.g., reflected by an increased release of sugars, amino acids, or inorganic ions from leaves towards leaf senescence [13, 14], which in turn affects growth and competition of phyllosphere-associated microorganisms and thus their successional dynamics [15–17]. These processes are central to phyllosphere community assembly, which is typically dominated by stochastic processes [18, 19], including the limitation of bacterial movement through physical, biological, or environmental constraints (dispersal limitation; [20]), or random changes in bacterial community composition due to replication, lysis and colonization (drift) [21].

The phyllosphere receives bacteria from various sources. These include atmospheric sources through air-borne inputs such as precipitation and dust particles [22, 23], while herbivorous and pollinator insects constitute another import vector [15]. In tree canopies, incoming rainfall is partitioned into throughfall, stemflow and interception loss [24]. Throughfall is rich in nutrients (C, N and P) [25] and may harbour up to 100 times more bacteria than rainwater [26]. Consequently, throughfall is likely to act as central vector in the translocation of microorganisms, and translocation may be taxon-specific, as the ability of bacteria to remain attached or be washed-off from leaf surfaces depends on their ability to form surfactants [27] or biofilms [28, 29]. Yet, these translocation processes have only rarely been studied, and observations were typically confined to collecting throughfall and the transported microbial communities below the canopy [12, 30, 31], lacking insight into the relevance of throughfall for within canopy distribution processes.

Here, we propose that phenology-driven changes in plant host properties and transport of microorganisms within the canopy via throughfall are key mechanisms driving canopy microbial diversity and its spatio-temporal variation in a temperate forest. We leveraged the infrastructure of the Leipzig canopy crane facility, installed in a floodplain hardwood forest, which allows full access to tree canopies of up to 35 m height, and we employed a novel approach of throughfall sampling within tree crowns at different height levels. Combined with sampling of the phyllosphere, this approach enabled us to assess vertical patterns of phyllosphere communities and their potential connection by throughfall-mediated transport processes. Focusing on three common tree species, *Quercus robur* L. (English oak), *Fraxinus excelsior* (European ash) and *Tilia cordata* MILL (Winter linden), we followed bacterial communities in phyllosphere versus throughfall across early (May), mid (July) and late (October) phenological stages at three different height levels within the canopy - top, mid, and bottom. We aimed to (i) disentangle the controls of tree species identity and canopy position on the phyllosphere microbiome across different phenological stages, (ii) follow introduction and succession of bacterial taxa and quantify the relative importance of different community assembly mechanisms, and (iii) evaluate the contribution of throughfall-mediated bacterial transport to phyllosphere microbiome patterns.

## Methods

### Site description

The sampling site is located in Burgaue, Northwestern part of Leipzig (Saxony, Germany) on the floodplains of the Elster, Pleisse, and Luppe rivers. The sampling site experiences a continental climate with the mean annual temperature of 8.4 °C and mean annual precipitation of 516 mm [32]. Annual precipitation in 2021 was 539 mm [33], making it slightly wetter than the long-term average. The Canopy Crane site consists of 18 different tree species and around 800 tree individuals in an area spanning 1.65 km^2^ [34]. The most abundant tree species in the forest are European ash (*Fraxinus excelsior* L.) and English oak (*Quercus robur* L.) [35], which are also the focal trees of this study along with *Tilia cordata* MILL (Winter linden). Hereafter, the tree common names are simply referred to as oak, ash and linden throughout the manuscript. The study area represents a mature old-growth forest with diameter at breast height (DBH) values of the sampled individuals ranging from 55 to 136 cm for oak, 68.7 to 84 cm for ash and 71 to 81 cm for linden. (Table S1).

### Sample collection and processing

**Fig. 1:**
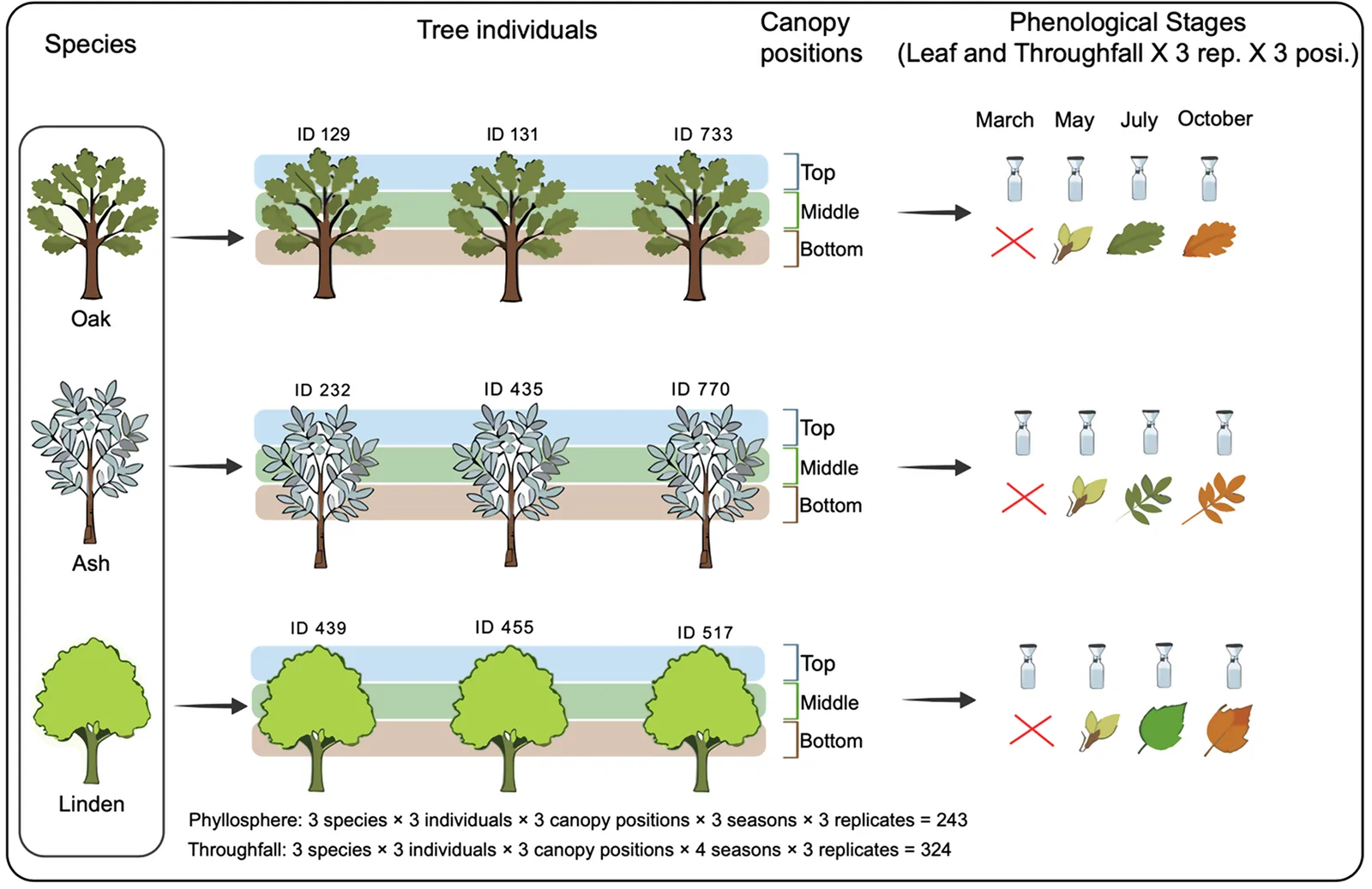
Study design and sampling strategy. Leaf and throughfall samples were collected from three individuals of oak, ash and linden trees at three canopy positions (Top, middle and Bottom) in three replicates per position during May, July and October, representing early, mid and late phenological stages respectively. Throughfall samplers were installed at the respective canopy positions 14 days prior to the day of sampling. Additional throughfall samples were also collected in March, when the trees were leafless

Leaf samples were collected from three individuals of each oak, ash and linden trees, at three different time points during the vegetation period, representing an early phenological stage directly after leaf sprouting (May), a mid-phenological stage in July and a late phenological stage in October 2021, when leaves showed clear signs of senescence (Fig. 1). In addition, to follow potential vertical changes in phyllosphere microbiome composition across the tree crowns, we sampled the top, middle and bottom canopy positions with each three spatial replicates at fixed positions. Where canopy structure and accessibility by the crane allowed, these three spatial replicates were distributed around the canopy, so that potential effects caused by locations facing different directions were included in the data variability among the three spatial replicates. The height of the positions for leaf sampling depended on the canopy architecture of the individual trees and varied from 26 to 34 m for the top position, 22.5–29.5 m for the mid position, and 19.5–27 m for the bottom position (Table S1). The different canopy positions were accessed using a gondola operated by a crane with a maximum sampling height of about 35 m. At each sampling position, approximately ten leaves were randomly collected from different twigs which were approximately 50 cm apart from each other using sterile scissors and were transferred to autoclaved polypropylene bottles. Leaves were always collected from the outside of the canopy. Throughfall samples were collected at the same time points as leaf samples and additionally in March 2021 (hereafter referred as March-throughfall). Throughfall collectors (sterile polypropylene bottles) with a funnel size of 113 cm^2^ surface area were deployed close to the locations of phyllosphere sampling (top, middle and bottom) within the canopy, at the same height levels as specified above, and exposed for a period of 14 days until throughfall samples were collected. Samples of rainwater which had not been in contact with the tree crowns were collected above the forest canopy by placing sterile polypropylene bottles on top of the crane (referred as “crane top”). Our sampling design allowed the collection of both dry deposition and rainwater-associated transport of microorganisms as well as the integrated sampling of multiple rain events during the exposure period. As sampling directly after single rain events was not possible with the infrastructure used, we cannot entirely rule out that growth of microorganisms in the throughfall containers may have led to a slight overestimation of throughfall bacterial abundances. Both leaf and throughfall samples were transported in a cooled condition with ice packs and stored at 4 °C upon arrival to the lab.

### Sample processing, DNA extraction and amplicon sequencing

For detachment of leaf-associated epiphytic bacteria, leaf samples were immersed in 250 ml of sterile suspension buffer (0.15 M NaCl, 0.1% Tween 20) in the collection bottles. These bottles were subjected to brief sonication twice for 1 min at 10% intensity, followed by shaking for 20 min at 100 rpm at room temperature to enhance the detachment of bacterial cells from the surface of the leaves. The resulting suspensions were filtered through sterile 0.22 μm pore size PES (polyethersulfone) membrane filters (Supor, Pall Corporation) to collect the bacterial cells. The remaining leaves were used for determination of dry weight after drying at 50 °C for one week. Throughfall samples were directly filtered through the same filter type. Filters were stored at − 80 °C until DNA extraction.

DNA was extracted from the filters using the DNeasy PowerSoil Pro Extraction kit (Qiagen) following the manufacturer’s protocol. We used the primers Bakt_0341F [36] and Bakt_0799R [11, 37] for the amplification of the V3-V4 region of bacterial 16 S rRNA genes, with the reverse primer being designed in order to avoid the amplification of chloroplast-related 16 S rRNA genes. The PCR amplification, paired–end library preparation and amplicon sequencing (2 × 300 base pairs; v3 chemistry) were carried out using an Illumina MiSeq sequencer based on a protocol described by [38]. The demultiplexed sequence files from this study were deposited in the European Nucleotide Archive (ENA) (accession number: PRJEB109949 and PRJEB109950). Quantitative PCR was performed using the primer combination Bakt_0341F [36] and Bakt_0799R [37] under the following cycling conditions: 95 °C for 10 min, followed by 45 cycles of 95 °C for 30 s, 53 °C for 60 s, and 72 °C for 40 s, followed by melting curve analysis. qPCR was performed using Brilliant II SYBR Green QPCR Master mix (Agilent Technologies) on a BioRad CFX96 platform (BioRad). PCR and qPCR reactions included non template controls using PCR grade water.

### Sequence analyses

We analysed raw sequence reads from rainwater (“crane top”), phyllosphere and throughfall samples using the Divisive Amplicon Denoising Algorithm 2 v1.26 dada2 R package [39]. We performed primer removal (*truncLeft(17*,*21*)), trimming (*filterAndTrim*; truncLen = c(280, 220)), error learning (*learnErrors*, *maxEE(2*,*4)*), denoising (*dada*), amplicon sequence variant (ASV) inference (*dada*), merging (*mergePairs*), table generation (*makeSequenceTable*), removal of chimeric reads (*removeBimeraDenovo*) and classification (*assignTaxonomy* using silva_nr99_v138.1 and silva_species_assignment_v138.1 databases) using dada2. The final count table for the crane top, phyllosphere and throughfall consisted of 10, 196 and 319 samples, respectively. Crane top, phyllosphere and throughfall count tables were rarefied to 19,780, 4217 and 4441 reads per sample, respectively based on the sample with lowest number of sequence counts. The count tables were further used for cell abundance estimations based on the following calculation, [relative abundance/average 16 S rRNA operon number (at family level)] X 16 S rRNA gene abundance data determined by qPCR. Briefly, the relative abundance values were divided by the family level mean 16 S rRNA operon numbers (https://rrndb.umms.med.umich.edu/), then multiplied with qPCR abundance data. The ASVs were categorised into abundant (> 0.1%), intermediate (0.01 to 0.1%) and rare (< 0.01%) [40] based on their relative abundance in the phyllosphere communities. ASVs that repeatedly occurred in consecutive phenological stages were referred to as ‘successional’ ASVs, while the unique ASVs were referred to as ‘newly introduced’ ASVs. The number of common and unique ASV present across phenological stages (early–mid and mid–late phenological stages) and vertically (top–mid and mid–bottom canopy positions) were assessed to understand the phenological and throughfall mediated vertical transfer associated changes, respectively.

### Functional prediction using PICRUSt2 analysis

Estimated cell abundances of bacterial ASVs, calculated as described above, were used for functional prediction using PICRUSt2 (Phylogenetic Investigation of Communities by Reconstruction of Unobserved States) [41]. In brief, the Kyoto Encyclopedia of Genes and Genomes (KEGG) ortholog (KO) gene families were predicted through the picrust2_pipeline.py function with the option of skipping operon normalisation (--skip-norm), since the input data was already copy number normalized. The assignment of KO families to various KEGG levels was carried out using the mapping file from PICRUSt2 Github (accessed on 14.05.2025). Pathway abundances (KEGG level 3) were compared between tree species and across phenological stages and tested for significance using Kruskal–Wallis and Dunn’s pairwise test.

### Community assembly process analysis

The assembly process analysis was carried out based on absolute abundance data using the phylogenetic bin–based null model analysis (iCAMP) R package [42], which was developed based on a previous study [43] to quantify the relative importance of ecological processes (deterministic: homogeneous selection (HoS) and heterogeneous selection (HeS), stochastic: dispersal limitation (DL), homogenizing dispersal (HD) and drift (DR)) governing phyllosphere microbiome community assembly across the phenological stages. Absolute abundance data were divided by a factor of 23.5 prior to analysis to avoid computational issues associated with large numbers. Briefly, phyllosphere ASVs were clustered into bins based on their phylogenetic similarities. These bins were subjected to an abundance–based null model analysis to calculate the beta net relatedness index and Raup–Crick through 999 pairwise iterations to infer the relative importance of deterministic (HoS: βNTI < -1.96, and HeS: βNTI > 1.96) and stochastic (|βNTI| < 1.96) (HD: RC < − 0.96; DL: RC > 0.96; DR: |RC| < 0.96) ecological processes governing bacterial community assembly in the phyllosphere.

### Statistical analyses and visualization

Principal coordinate analysis (PCoA) was performed using the vegan R package [44] in R to analyze the clustering of samples in the ordination space across phenological stages. Singletons were removed before performing PCoA analysis. Permutational ANOVA (PERMANOVA) analysis (999 iterations) was performed to assess the contribution of phenological stages (month), tree species identity (host), and canopy position (position), as well as their two-way interactions (month × host, host × position, and month × position), on bacterial community structure using the vegan R package. Phenological stage-wise PERMANOVA tests were also conducted to assess the relative contribution of tree species and canopy position to bacterial community variability for each month separately. Tree individual was included as a random effect in the PERMANOVA analysis to account for the dependency among repeated observations from the same tree. Pairwise Bray–Curtis dissimilarity values between months were used to assess relative similarity in bacterial community composition, with lower dissimilarity values indicating greater similarity. Linear mixed-effects model (LMM) analyses were performed for analysing the effect of phenological stages, tree species identity, and canopy position on Shannon’s diversity H index, total abundance, family level abundance and throughfall bacterial flux. In the model, the phenological stages, tree species, canopy positions and their two-way interactions were kept as fixed effects, while repeated sampling of the same trees across phenological stages was included as a random effect. The main and interaction effects were assessed using LMMs. Estimated marginal means and Tukey-adjusted pairwise comparisons were calculated using the emmeans R package. Significant pairwise differences were indicated on the boxplots using different letters. The bacterial abundance and throughfall bacterial flux data were log_10_-transformed to reduce skewness and improve homogeneity of residual variance. The model assumptions were assessed using residual diagnostics and multicollinearity was assessed using variance inflation factors (VIFs). Boxplots were generated using the ggplot2 R package. Venn diagrams were used to understand the percentage of successional and unique ASVs across phenological stages using eulerr R package. Heatmaps with hierarchical clustering for ASVs (based on Euclidean distances) were created using the pheatmap R package to follow abundance changes (log transformed) across the phenological stages. Key ASVs which increased or decreased in terms of abundance across the phenological stages were identified based on linear discriminant score (LDA) using linear discriminant analysis effect size (LEfSe, microbiomeAnalyst). Key ASVs that preferentially occurred in crane top samples (rainwater), in the phyllosphere or in throughfall were analysed by comparing the relative abundances of common ASVs between any two selected groups (crane top vs. throughfall at the top canopy position or phyllosphere vs. throughfall at all three canopy positions) using LEfSe analysis. The throughfall bacterial flux was calculated as per sampling event by factoring the estimated cell abundances per L, rainwater/throughfall volume during the sampling period, and surface area of the collectors. The KEGG assignment resulting from PICRUSt2 analysis was carried out at category (level 1), sub-category (level 2) and pathway (level 3) levels. KEGG pathway abundance changes at level 3 were analyzed pairwise (early–mid and mid–late phenological stages) for each tree species using Kruskal–Wallis and Dunn’s pairwise test, *P* < 0.05 using the stats R package. The KEGG pathway abundance (log_10_–transformed) change across phenological stages was visualised using the pheatmap package in R.

## Results

### Changes in phyllosphere bacterial community patterns and abundance across early, mid, and late phenological stages

**Fig. 2:**
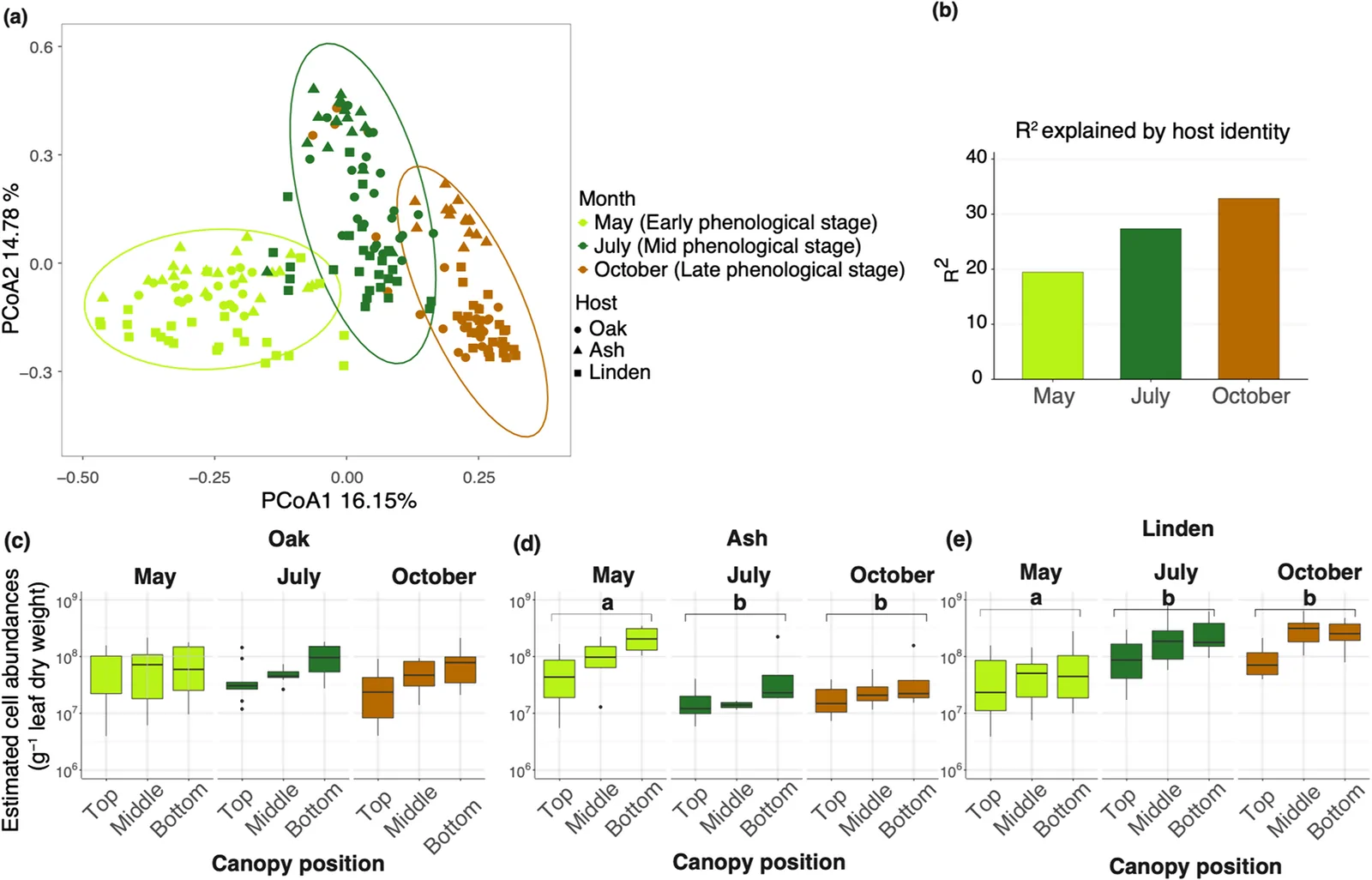
Principal coordinate analysis of phyllosphere bacterial communities on oak, ash, and linden across phenological stages. (**a**) Sample-based ordination plot for phyllosphere bacteria (operon normalised) generated from amplicon sequence variants (ASV) compositions. Ellipses represent 95% confidence intervals for the tested factor variable (i.e. Phenological stages). (**b**) Community variation (R^2^) (0–100) explained based on host identity according to phenological stages, May (Early), July (Mid) and October (Late phenological stages). (**c**-**e**) Estimated cell abundances of phyllosphere bacteria across tree species and canopy positions. Different letters in the boxplots indicate significant differences across phenological stages within tree species based on the linear mixed-effects model and Tukey-adjusted pairwise comparisons (*P* < 0.05). The model included tree species, phenological stage, canopy position, their two-way interactions, and tree individual as a random effect. The absence of letters point to statistically non-significant results

Phenological stage was the most important factor driving phyllosphere bacterial community composition, as it explained 22.59% of community variability, followed by tree species identity (12.79%). In contrast, canopy position (2.15%) constituted only a minor contribution to community variability (Fig. 2A, Table S2). Interactive effects between phenological stage and tree species identity explained another 6.7% of variability, while contributions of interactive effects between tree species identity and canopy position, or phenological stage and canopy position, contributed each less than 2% (Table S2).

Phyllosphere communities sampled at early, mid and late phenological stages were clearly distinct (pairwise PERMANOVA, *P =* 0.001 for all comparisons), with a slightly higher similarity between bacterial communities of the mid and late phenological stages (July–October mean Bray–Curtis dissimilarity = 0.662) compared to the early phenological stage (May–July = 0.751; May–October = 0.754). When analyzing community drivers separately for each phenological stage, tree species identity explained most of the bacterial community composition variability with a gradually increasing effect from the early (19.47%) to mid (27.36%) and late (32.86%) phenological stage (Fig. 2B, Table S2). The contribution of canopy position was similar across the phenological stages and ranged from 4.99% (May) to 5.89% (October) (Table S2).

The phyllosphere Shannon’s diversity index (H′) varied significantly with phenological stage and canopy position. Diversity was significantly higher during the mid and late phenological stages than during the early stage, whereas the mid and late stages did not differ significantly from each other (Fig. 2C–E; Additional file 2). Despite the rather small effect of canopy position on phyllosphere community composition, phenological stage and canopy position significantly influenced Shannon’s diversity index (H’) (*P* < 0.001), which was lower in the top canopy position compared to the middle and bottom positions (Fig. S1; Additional file 2). Although tree species did not have a significant main effect, the significant tree species × canopy position interaction (*P =* 0.009) indicated that the effect of canopy position differed among tree species (Table S3; Additional file 2).

Estimated phyllosphere bacterial abundance was significantly affected by canopy position (*P* = 0.0065) but not by tree species or phenological stage as single factor (*P* = 0.346 and *P =* 0.157, respectively) (Table S3; Additional file 2; Additional file 3). The significant interactive effect of tree species × phenological stage (*P* < 0.001) indicated that temporal patterns in bacterial abundance differed among tree species. In contrast, the significant effect of canopy position was not further modulated by tree species or phenological stages (tree species × canopy position, *P* = 0.639; phenological stage × canopy position, *P* = 0.597).

### Compositional changes and successional transfer across phenological stages

Bacteria belonging to 20 phyla, 203 families, and 527 genera were detected in the phyllosphere samples of this study. Ninety–five percent of the total reads belonged to the phyla Proteobacteria (68%), Bacteroidota (14%) and Actinobacteriota (13%), with *Sphingomonadaceae*, *Beijerinckiaceae*, *Pseudomonadaceae*, *Erwiniaceace*, *Oxalobacteraceae*, *Acetobacteraceae*,* Microbacteriaceae* and *Kineosporiaceae* representing the most abundant families. The relative abundance of top 20 bacterial families ranged from 74.6 to 96.3% of the total community (Fig. S2), with patterns differing across the phenological stages and tree species.

*Beijerinckiaceae* and *Erwiniaceae* were selected for a more detailed family-level abundance analysis because of their functional relevance as plant growth-promoting/biocontrol bacteria and plant pathogens, respectively, and also due to contrasting trends of these taxa between the early and mid-phenological stages. The estimated cell abundances of bacteria affiliated with *Beijerinckiaceae* increased and *Erwiniaceae* decreased from the early to mid-phenological stages across the three tree species, whereas their patterns from the mid to late phenological stages varied among tree species, indicating tree species-specific seasonal trajectories (Fig. S3; Additional file 2). The estimated cell abundance of bacteria affiliated with *Beijerinckiaceae* was significantly influenced by phenological stage (*P* = 0.0039) and canopy position (*P* = 0.0101), with a significant tree species × phenological stage interaction (*P* = 0.0032) (Table S3). Similarly, *Erwiniaceae* abundance was significantly influenced by phenological stage (*P =* 0.0423), with a significant tree species × phenological stage interaction (*P* < 0.001), but without a significant effect of canopy position (*P* = 0.878) (Table S3).

**Fig. 3:**
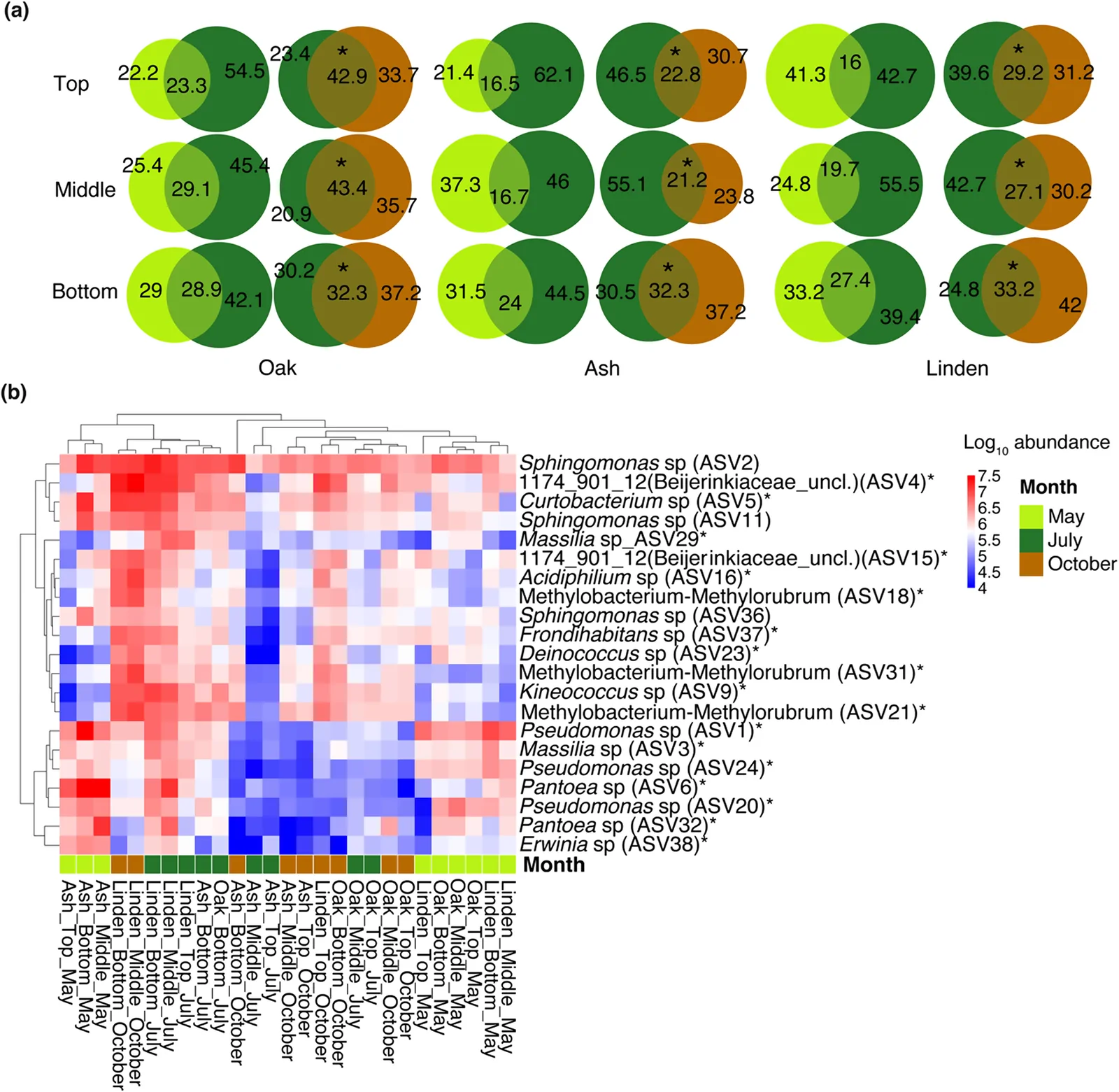
Top and shared amplicon sequence variants across phenological stages in the phyllosphere. (**a**) Venn diagram showing the shared and unique amplicon sequence variant (ASV) percentages among May, July and October. “Top”, “Middle” and “Bottom” denote canopy positions. May, July and October indicate phenological stages. * indicates statistical significance of shared ASV percentages between May-July and July-October based on two-proportion z-test (*P* < 0.05). (**b**) heatmap showing the top phyllosphere amplicon sequence variants (ASVs). The colour intensity of the palette indicates the log_10_ estimated cell abundances from low (blue) to high (red). * denotes differentially abundant (*P* < 0.05) phyllosphere ASVs based on LEfSe analysis

To assess the successional transfer of ASVs across the phenological stages, we analyzed the shared ASVs of the phyllosphere communities between early and mid or mid and late phenological stages. The fraction of shared ASVs was typically higher between the mid and late phenological stage (22–43%) compared to the transition from the early to the mid phenological stage (16–29%) at all canopy positions and across all three tree species (Fig. 3A). The cumulative relative abundance of successional ASVs, defined as ASVs that repeatedly occurred in consecutive phenological stages, was higher (50–92%) than that of newly introduced ASVs (8–50%) for both the July and the October communities (Fig. S4 and Table S4). In turn, the relative abundance of newly introduced ASVs was higher for the mid (16–50%) compared to the late (8–13%) phenological stage (Fig. S4). The successional ASVs associated with oak and linden mostly belonged to the category of abundant taxa (> 0.1%) (23–49% of all ASVs), while for ash, successional ASVs were mostly represented by ASVs of the category of rare (< 0.01%) or intermediate taxa (< 0.1% and > 0.01%; 39–78% of all ASVs) (Fig. S5 A and Table S4). Newly introduced ASVs mostly belonged to the category of rare ASVs (< 0.01%) (35–91%), irrespective of phenological stage or tree species (Fig. S5 B).

The heatmap of the distribution patterns of the 20 most abundant ASVs showed a more distinct clustering of samples from the mid to late compared to the early phenological stage, mirroring the observations of the shared ASV (%) trends (Fig. 3B). When moving from early to late phenological stages, abundance of unclassified *Beijerinckiaceae* increased, while abundances of potential plant pathogens, such as *Erwinia* sp. (ASV38), *Pantoea* sp. (ASV32) and *Serratia* sp. (ASV33) decreased (Fig. 3B; Additional file 4) (LEfSe, *P* < 0.05). Specific abundant ASVs were newly introduced only during mid and late phenological stages across tree species, or appeared specific to oak, ash, or linden (Table S5).

### Community assembly processes in the tree phyllosphere during mid and late phenological stages

**Fig. 4:**
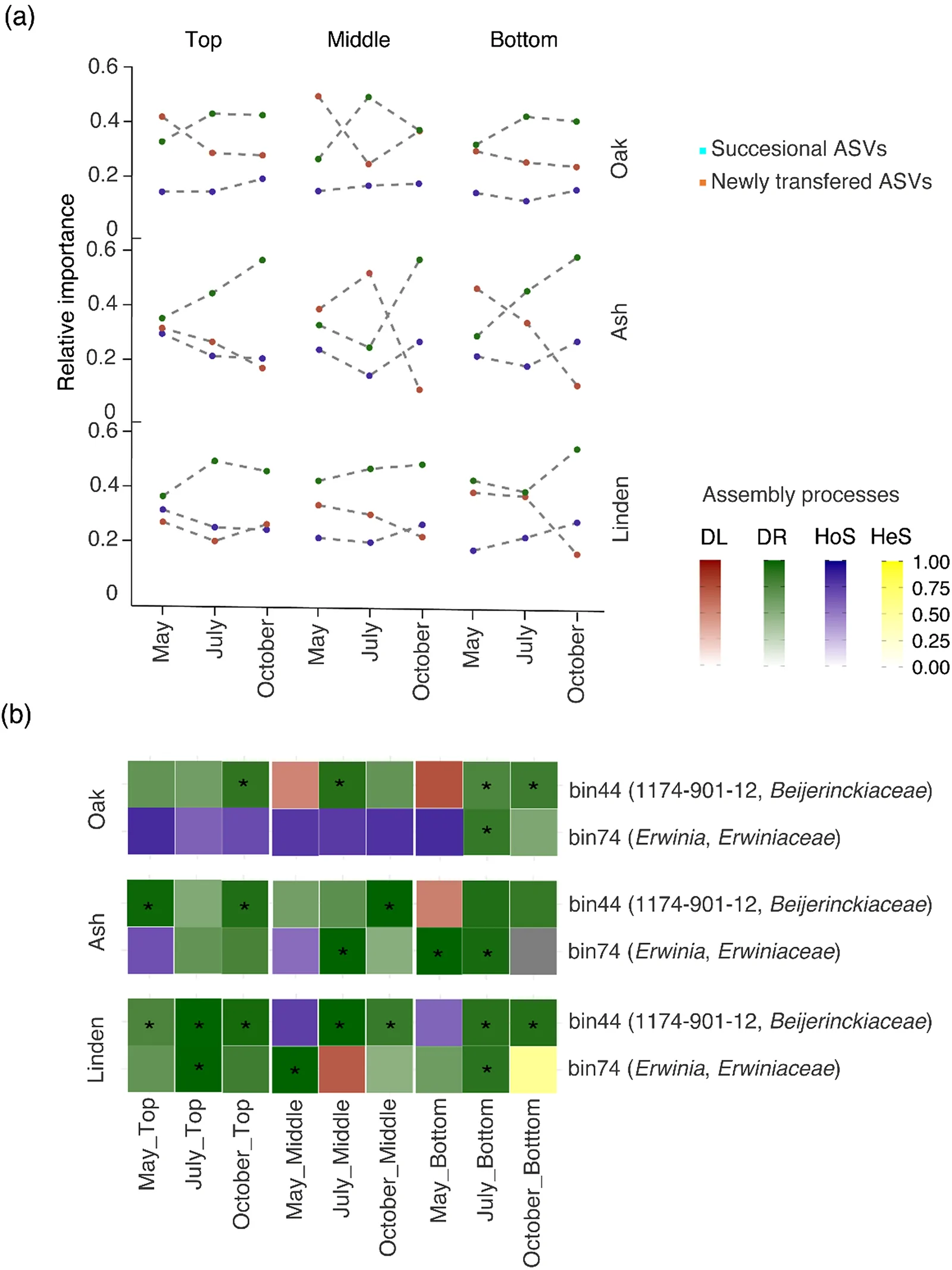
Relative importance of assembly processes and key ASVs across canopy positions, phenological stages and tree species. The relative importance (0 to 1) of assembly processes across canopy positions, phenological stages and tree species for (**a**) overall and (**b**) key bins. * denotes statistical significance (*P* < 0.05) of the process contributed by the respective bins as per iCAMP analysis

Community assembly process analysis was performed to understand the relative importance of deterministic (homogenous selection and heterogenous selection) and stochastic processes (dispersal limitation, drift, and homogenizing dispersal) that govern the assembly of the phyllosphere bacterial communities. Drift (25–54%), dispersal limitation (10–52%), and homogeneous selection (15–32%) were the dominant assembly processes across months and canopy positions (Fig. 4A) (Additional file 6.xlsx). A total of 126 bins representing ASVs that are phylogenetically related and putatively share similar ecological functions were detected in this study. The 15 top bins accounting for 62% of the total relative abundance were dominated by ASVs affiliated with *Sphingomonas*, unclassified *Beijerinckiaceae*,* Pseudomonas*, *Methylobacterium–Methylorubrum*,* Rosenbergiella*, *Curtobacterium*, *Pantoea*, *Massilia*, and *Erwinia*) (Additional file 7).

At an early phenological stage, community assembly was dominated by dispersal limitation (32–47%), drift (29–38%), and homogenous selection (17–30%), which shifted towards drift (25–58%) during the mid and late phenological stages for oak and ash (Fig. 4A). For linden, the drift–based community assembly dominated across canopy positions and phenological stages (37–54%) (Fig. 4A). The contribution of successional and newly introduced ASVs towards the three dominant processes (homogenous selection, drift and dispersal limitation) showed that the successional ASVs were the major driver of drift, while the newly introduced ASVs were linked to dispersal limitation (Fig. 4A). As the communities transitioned from early to mid and late phenological stages, the relative importance of dispersal limitation decreased which was linked to decreasing abundances of *Erwiniaceae* (bin 74), while the relative importance of drift mediated by *Beinjerinckiaceae* (bin 44) increased (Fig. 4B; Additional file 6).

### Functional potential of tree phyllosphere bacteria across the phenological stages

**Fig. 5:**
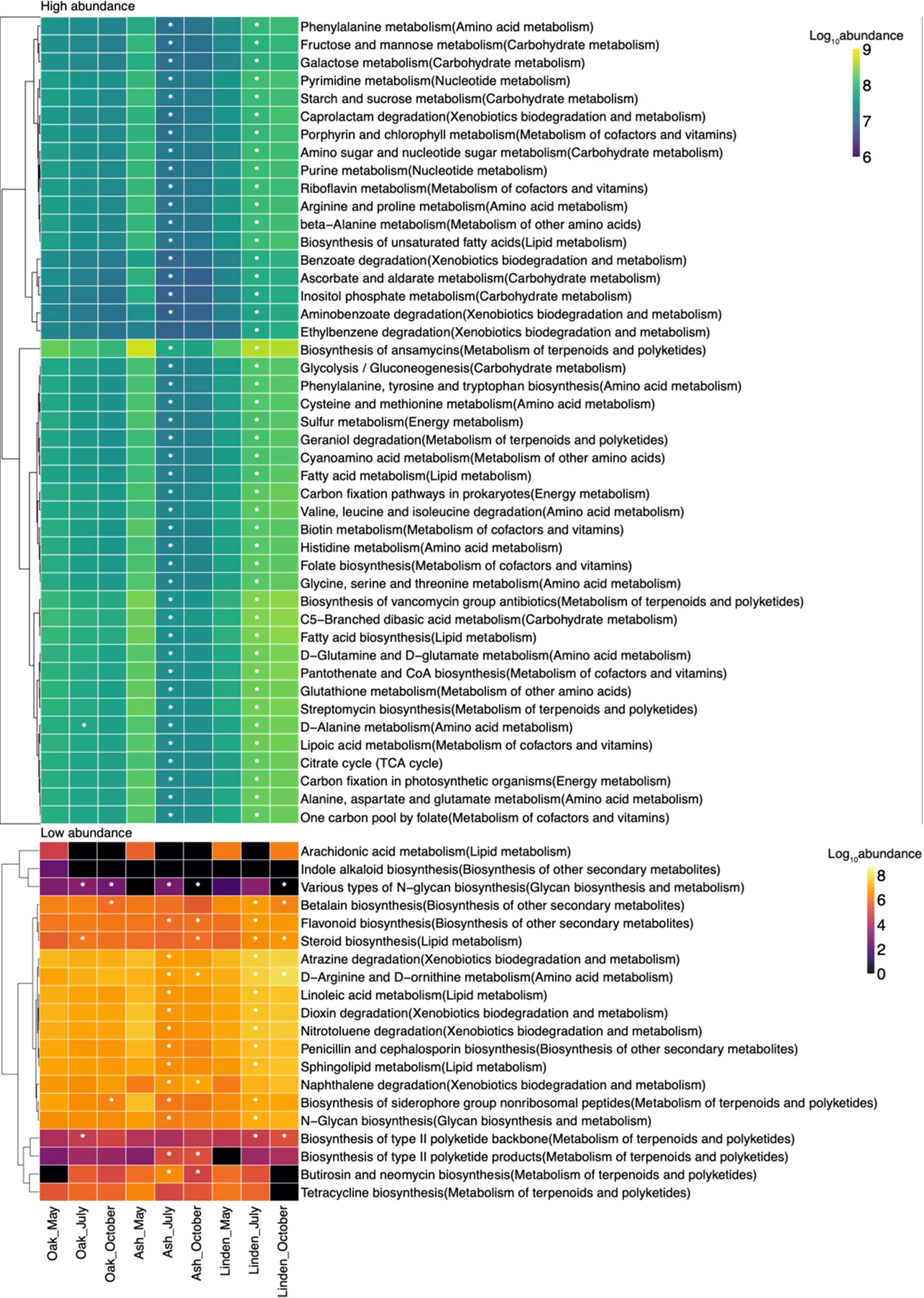
Heatmap showing KEGG Level_2 and Level_3 pathway abundances at phenological stage and tree species level. The Euclidean based hierarchical clustering was applied for rows (KEGG pathway abundances). Pathways were separated into higher- and lower-mean-abundance groups based on the 30th percentile of mean pathway abundance across all samples. The colour intensity of the palette indicates the log transformed pathway abundances calculated from estimated cell abundances (g^− 1^ dry leaf) from low (blue) to high (red). * in July and October indicates statistical significance (Kruskal–Wallis and Dunn’s pairwise test, *P* < 0.05) when compared with their respective preceding phenological stage

Functional potential analysis of phyllosphere bacteria identified 65 level 3 KEGG pathways associated with the category “metabolism”, of which the majority remained unchanged between mid and late phenological stages (Kruskal–Wallis and Dunn’s pairwise test, *P* > 0.05) across all tree species (64 in oak, 58 in ash, and 60 in linden)(Fig. 5; Additional file 8). For all three tree species, KEGG pathways belonging to type II polyketide synthase (PKS) and steroid and sphingolipid biosynthesis pathways belonging to lipid metabolism were enriched during mid and late phenological stages (Kruskal–Wallis and Dunn’s pairwise test, *P* < 0.05). Additionally, we observed an increase in flavonoid, butirosin and neomycin biosynthesis pathway abundances (ash), flavonoid biosynthesis pathways (linden), and non-ribosomal peptide pathways (oak) during the mid and late phenological stages (Kruskal–Wallis and Dunn’s pairwise test, *P* < 0.05).

### Bacterial communities in throughfall

**Fig. 6:**
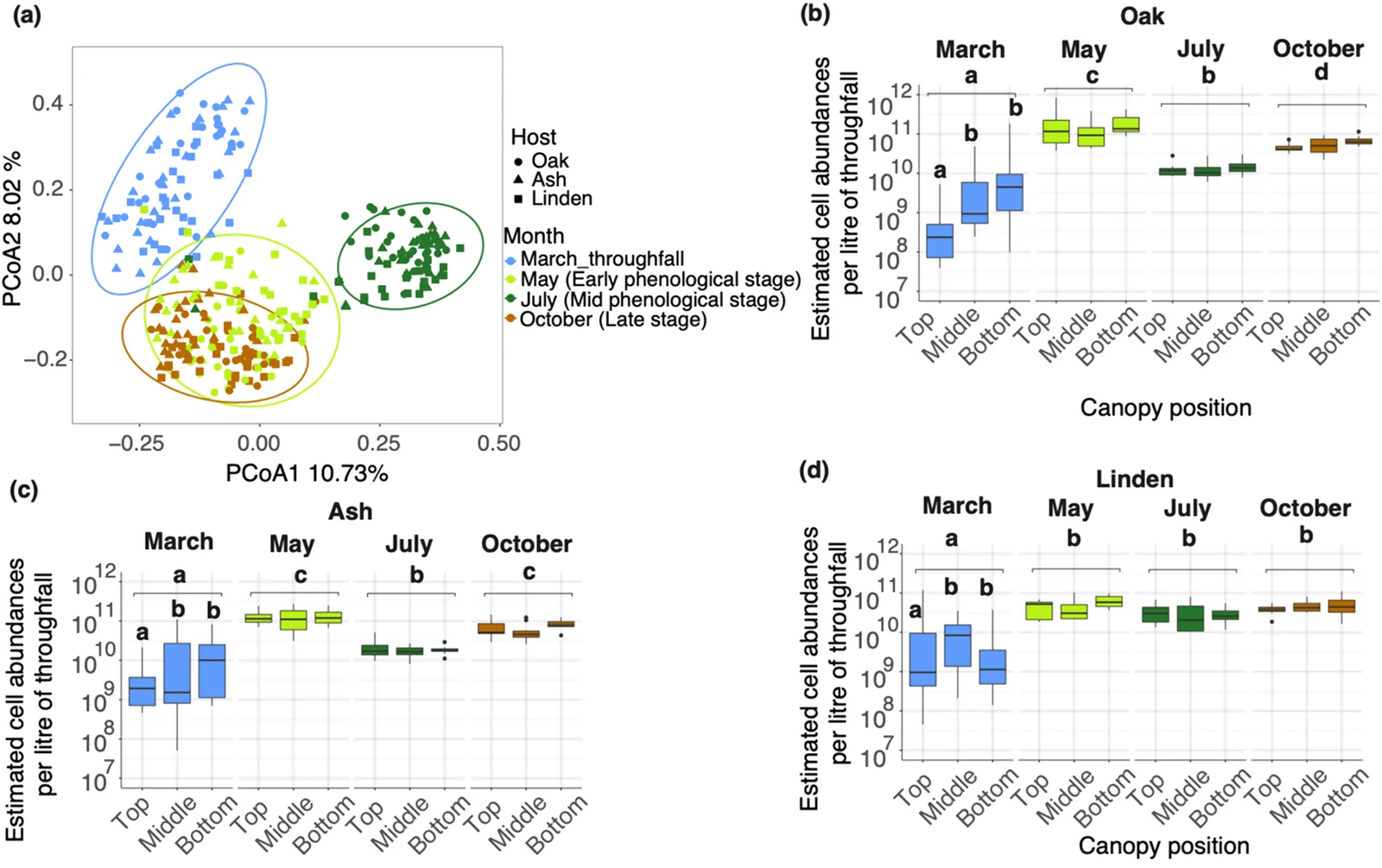
Principal coordinate analysis of throughfall bacterial communities across oak, ash, linden, early, mid, late phenological stages. (**a**) Sample-based ordination plot generated from amplicon sequence variant (ASVs) (operon normalised) compositions of throughfall bacteria. Ellipses represent 95% confidence intervals for the tested factor variable (i.e. phenological stages and throughfall from leafless March month), (**b**-**d**) Estimated cell abundances of throughfall bacteria across tree species and canopy positions. Different letters in the boxplots indicate significant differences across phenological stages and within canopy positions based on the linear mixed-effects model and Tukey-adjusted pairwise comparisons (*P* < 0.05). The model included tree species, phenological stage, canopy position, their two-way interactions, and tree individual as a random effect. The absence of letters point to statistically non-significant results

Similar to the phyllosphere communities, phenological stages explained 19.98% of the variation in bacterial communities transported by throughfall, followed by the influencing factors tree species (4.38%) and canopy position (2.22%) (Fig. 6A, Table S2). Throughfall bacterial communities collected in March before leaf sprouting were distinct from the other phenological stages.

The Shannon’s diversity index (H′) was highest for throughfall collected in early and mid-phenological stages across all tree species (Fig. S6). Throughfall Shannon’s diversity (H′) varied significantly with phenological stage (*P* < 0.001) and canopy position (*P* < 0.001) (Fig. S6; Table S3). Estimated bacterial cell abundances in throughfall were lowest during March and increased during the subsequent phenological stages, with a significant effect of phenological stage (*P* < 0.001) (Fig. 6B–D; Table S3). Canopy position also significantly affected the throughfall estimated bacterial abundances (*P* = 0.0031), while significant tree species × phenological stage and phenological stage × canopy position interactions (*P* < 0.001) indicated that seasonal abundance patterns varied among tree species and canopy positions (Fig. 6B–D; Table S3; Additional file 2). In order to estimate the total flux of bacteria being transported by rainwater through the canopy system, we calculated bacterial fluxes per m^2^ and sampling event. Throughfall bacterial flux varied significantly with phenological stage (*P* < 0.001) and was highest in early (7.25 × 10¹²) compared to mid (1.31 × 10¹²) and late (8.36 × 10¹¹) phenological stages. Across the three tree species, bacterial flux was highest in the top canopy position, with mean values of 9.77 × 10¹², 1.80 × 10¹², and 8.98 × 10¹¹ in early, mid, and late phenological stages, respectively, compared with lower fluxes in the middle and bottom canopy positions (Fig. S7; Additional file 2). Throughfall bacterial flux showed a significant tree species-dependent temporal pattern (tree species × phenological stage, *P* < 0.001), with the seasonal pattern also varying among canopy positions (phenological stage × canopy position, *P* = 0.0013) (Fig. S7; Additional file 2). The effect of canopy position was significant, with fluxes differing between the top, middle, and bottom positions (*P* < 0.001; Tukey-adjusted pairwise comparisons: top vs. middle, *P* = 0.048; top vs. bottom, *P* < 0.001; middle vs. bottom, *P* = 0.001). Across all linear mixed-effects models, VIF values were low (1.07–1.17), indicating negligible multicollinearity among the predictors and interaction terms (Additional file 2).

**Fig. 7:**
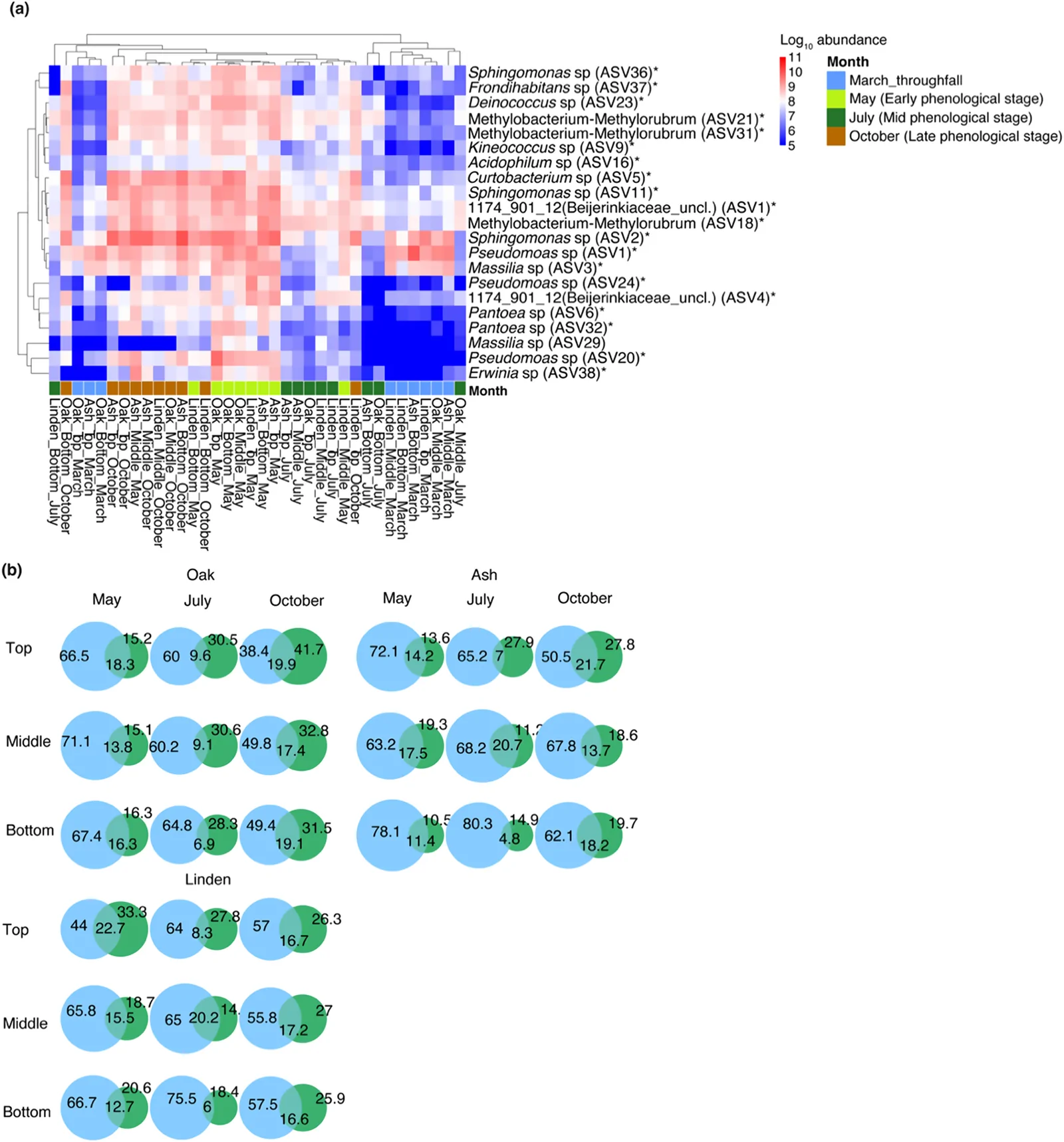
Top throughfall ASVs and shared ASVs (throughfall–phyllosphere) across tree species, phenological stages and canopy positions. (**a**) Heatmap showing top throughfall amplicon sequence variants (ASVs). The colour intensity of the palette indicates the log transformed estimated cell abundances from low (blue) to high (red). (**b**) Venn diagram showing the shared and unique amplicon sequence variant (ASV) percentages between throughfall and phyllosphere samples for early, mid and late phenological stages. March-throughfall indicates throughfall samples before the start of bud bursting. * denotes differentially abundant (*P* < 0.05) throughfall ASVs based on LEfSe analysis

The bacterial communities transported by throughfall consisted of families that were also commonly detected in the phyllosphere, including *Pseudomonadaceae*, *Oxalobacteraceae*, *Microbacteraceae*, *Beijerinckiaceae*, *Erwiniaceae*, *Rhizobiaceae* and *Spirosmaceae* (Fig. S8). *Oxalobacteraceae* and *Pseudomonadaceae* clearly dominated the throughfall communities in March before foliage emerged. While ASVs affiliated with *Sphingomonas*, *Pseudomonas*, and *Massilia* exhibited consistently high abundances in throughfall across all tree species and time points, relative abundances of ASVs affiliated with *Methylobacterium-Methylorubrum*, *Kineococcus*, *Acidiphilum*, *Curtobacterium*, *Pantoea*, and *Erwinia* increased only after the sprouting of leaves during the early phenological stage (Fig. 7A). Estimated throughfall-mediated vertical transfer pointed to a lower exchange of ASVs between throughfall and phyllosphere surfaces in the mid (5–21%) compared to the early (11–23%) and late phenological stages (13–26%) (Fig. 7B) (Kruskal–Wallis and Dunn’s pairwise test, *P <* 0.05).

**Fig. 8:**
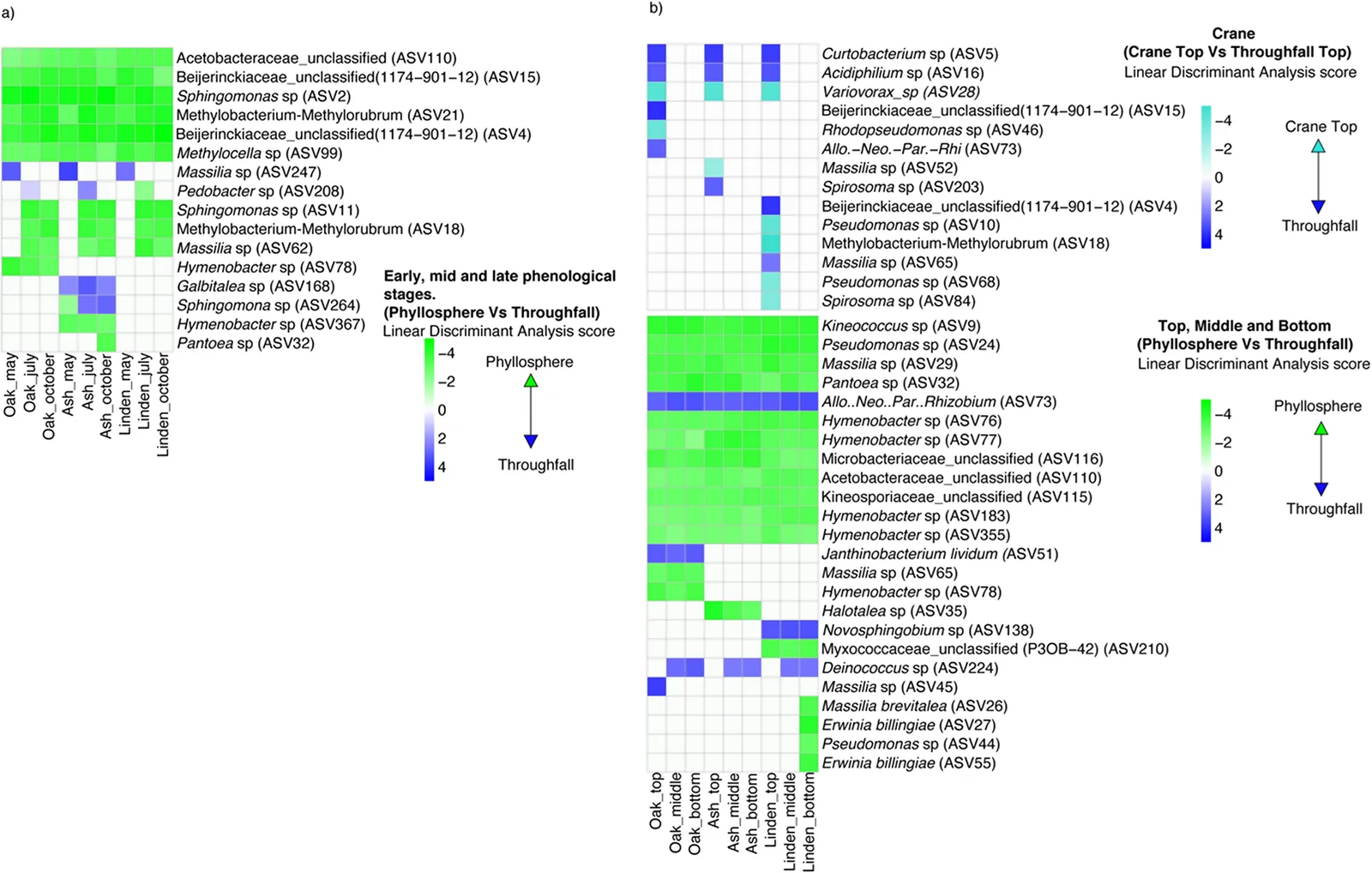
Successional and vertical trends of phyllosphere attachment and throughfall wash–off patterns of amplicon sequence variants. The relative abundances of shared ASVs between groups were analyzed to identify differential enrichment associated with (**a**) phenological stages (early, mid and late phenological stages) and (**b**) vertical canopy positions (crane top, top, middle, and bottom canopy positions). LDA scores were calculated using LEfSe (*P* < 0.05 and FDR < 0.05). For crane top vs. throughfall top comparisons, turquoise blue color indicates crane top enrichment, blue indicates throughfall wash-off at top position. For phyllosphere vs. throughfall comparisons, green color indicates preferential attachment, blue indicates preferential throughfall-mediated wash-off across all canopy positions

Bacterial taxa prone for either leaf attachment in the phyllosphere or wash-off during successional transfer (early, mid and late phenological stages) and vertical transfer (crane top, top, mid and bottom canopy positions) were identified based on LDA scores (LEfSe analysis) derived from the relative abundance of shared ASVs in throughfall versus phyllosphere (*P <* 0.05 and FDR < 0.05) (Fig. 8A; Additional file 5). A consistent higher prevalence in the phyllosphere was observed for ASVs affiliated with *Kineococcus* sp, *Pseudomonas* sp, *Massilia* sp, *Hymenobacter* sp, *Pantoea* sp., *Sphingomonas*, *Methylobacterium-Methylorubrum*, *Methylocella*, and other *Beijerinckiaceae*, *Microbacteriacae*, and *Acetobacteraceae* (Fig. 8B). In contrast, a higher tendency to get mobilized by throughfall was detected for ASVs affiliated with *Allo*-*Neo-Para-rhizobium* and *Deinococcus* across all tree species, or for *Janthinobacterium* and *Novosphingobium* only in association with oak or linden, respectively (Fig. 8B).

## Discussion

Our findings clearly demonstrated that phenological stage is a key driver of phyllosphere microbiome variability in a temperate hardwood forest, exceeding the effects of tree species identity. Similarly, phenology was previously recognized to play a key role in phyllosphere microbiome variability in evergreen (*Pinus* and *Sabina*) [45] and perennial (*Citrus* and *Acacia*) plants [8, 9]. While studies of temperate forests focusing on a single sampling time point primarily identified host species identity as the main drivers of phyllosphere bacterial communities [46, 47], Laforest-Lapointe et al. 2016 [10] confirmed the predominant role of tree species also when taking multiple samples throughout the vegetation period in a Quebec temperate forest. This contrasting finding to our study could be attributed to lower successional dynamics during a three-months observation period in the Canadian forest, or a stronger selection by the respective host tree species. In contrast to our earlier observations [11], canopy position explained only a minor part of phyllosphere microbiome variability. Yet, we present strong evidence that canopy-position dependent vertical trends of phyllosphere bacterial abundance are tightly coupled to throughfall-mediated within-canopy transport.

Early phenological stage communities after leaf sprouting were distinct, while phyllosphere bacterial communities became more similar and functionally redundant during mid–late phenological stages. The observed temporal community patterns may have been caused by combined effects of tree species specific factors and external factors that vary seasonally, such as leaf-visiting insects for pollination or herbivory [48], inputs of pollen and dust, and extent of sun exposure. From the tree host perspective, phenological stages are characterized by changes that synchronize with bud burst and leaf out at the early phenological stage to heightened metabolic activities during the mid phenological stages such as photosynthesis [49] and trace gas production [50]. Towards senescence, release of sugars, amino acids, or organic ions from leaves typically increases [13]. Of note, oak, ash, and linden differ in their leaf nutrient resorption and nutrient loss through leaching especially upon leaf senescence [51], providing one possible explanation why the effect of tree species on the phyllosphere microbial communities was the strongest at the late phenological stage. Throughout the vegetation period, the effect of tree species could also be driven by differences in pollination type and timing (oak, ash: wind pollinated; linden: insect-pollinated), leading to tree-species dependent differences in pollen-mediated microbial import or nutrient provision [52]. Similarly, heavy aphid infestations typically occurring on linden trees may additionally change the amount and spectrum of organic carbon compounds available to the phyllosphere microbiota [53].

In line with these phenology-driven changes, phyllosphere communities in July and October shared a larger fraction of successional ASVs compared to the fraction shared with the early phenological stage in May, suggesting a transition from bacterial communities which are mostly characterized by random colonization to communities that are more closely associated with tree host properties, as the vegetation period progressed. In fact, the fraction of phyllosphere bacterial community variability explained by tree species identity increased from 19.5% in May to 32.9% in October, indicating an increasing relevance of tree-species specific drivers with ongoing leaf maturation and senescence. Young leaves are known to have a higher nitrogen content [15, 16, 54]. Consequently, early bacterial colonizers likely occupied leaf buds and quickly depleted the available resources including nitrogen compounds through competitive exclusion [55], thereby repelling the colonisation by other bacteria during the early phenological stages. Increased availability of leaf-associated resources such as volatile organic compounds, sugars, or amino acids during mid and late phenological stages [13, 14, 56] likely increased phyllosphere niche diversity and enabled the growth, establishment, co-existence, and successional transfer of more taxa [55], resulting in the observed diverse phyllosphere bacterial communities of the mid and late phenological stages. The quantitative relevance of newly introduced bacteria was typically lower during the late phenological stage as the phyllosphere habitat became increasingly colonized and occupied.

The described phenology-driven changes in host plant physiology as well as seasonal immigration patterns of atmospheric, pollen- or insect-associated microorganisms are likely to have a strong effect on the competition between introduced and successional bacterial leaf colonizers. In turn, the balance between introduction, competition and persistence of microbial taxa may be crucial for maintaining necessary ecosystem functions at the respective phenological stages. While moving from early to mid-phenological stage, we noted a shift of bacterial communities from being dominated by putative plant pathogens (e.g. *Erwiniaceae*) to being dominated by bacterial taxa associated with plant growth promotion and biocontrol (e.g. *Beijerinckiaceae*) [57]. Low plant defense capabilities [17] are characteristic of plant physiological conditions at early phenological stages, which possibly favored the colonization by bacterial pathogens such as *Erwiniaceae*. The observed later decrease in the abundance of putative plant pathogens (*Erwinia* sp., *Pseudomonas* sp., *Pantoea* sp., and *Serratia* sp.) during the mid phenological stage likely occurred due to competitive exclusion by early colonizers [58, 59]. The higher abundance of putative steroid and secondary metabolite related pathways (Type II PKS, flavonoid and non-ribosomal peptide) indicates the importance of signalling molecules towards plant growth promotion [60] and antagonistic mechanisms in pathogen control [61], respectively, during the mid phenological stage.

Despite the increasing influence of tree species on phyllosphere communities as the vegetation period progressed, bacterial community assembly in the phyllosphere was mainly driven by stochastic processes, especially dispersal limitation and drift. In models of stochastic assembly, random factors or events, including lack of movement, colonization and extinction shape community composition over time [62]. Dispersal limitation dominated during the early phenological stage, likely due to the spatial separation of small leaf sprouts and the random nature of the colonization events. The early colonizers, especially pathogens (e.g. *Erwinia* sp), likely depleted the available space and resources during leaf budding through priority effects [58]. Similarly, previous work identified priority effects as a driving factor for heterogeneous phyllosphere bacterial communities, along with a dominance of dispersal limitation at an early phenological stage [58]. During mid and late phenological stages, new bacteria entered and shared the resources with successional bacteria (e.g. *Beijerinckiacea*e) through niche partitioning, which led to a shift of the dominant assembly process towards drift. Despite continued taxonomic turnover, the higher abundance of bacteria carrying out methylotrophic and metabolically diverse functions in the communities of the mid and late phenological stages resulted in functional redundancy [63], which has been reported both as a consequence as well as a potential driving factor for drift assembly [63, 64]. Functional redundancy was further reflected in the stable abundance patterns across predicted homeostasis pathways, e.g., carbohydrate and energy metabolism.

Assessment of rainwater-mediated bacterial transport into and within the canopy system allowed us to identify potential airborne import of microbes and taxon-specific mobilization patterns. In general, throughfall-mediated transport and mobilization resulted in a substantial flux of microbial cells through the tree crowns of up to 10^12^ cells per m^2^ per sampling event, which is in the same range as throughfall bacterial loads measured below the canopy in a previous study [31]. These findings support our hypothesis that rainwater plays a key role in the distribution of bacteria within the canopy, acting as an important facilitator of bacterial colonization but also detachment. Contact of throughfall with foliage resulted in an increase of transported bacteria by up to two orders of magnitude when comparing leafless (March) and foliated stage (May), or rainwater collected above the tree canopy to throughfall collected at the canopy top.

For both oak and linden, Shannon diversity and abundances of the phyllosphere bacteria were highest at the bottom position of the canopy, suggesting that throughfall-mediated wash-off and transport from above parts resulted in an increased bacterial import to the lowest position of the tree crown. In turn, throughfall-mediated mobilization was the most intense at the canopy top across all three tree species, coinciding with lowest phyllosphere bacterial diversity and abundance at this position. Consequently, reduced diversity and abundance at the canopy top might not only be a result of generally harsher conditions at this exposed position [65], but phyllosphere taxa might also be more prone to detachment under these conditions. This likely results in continuous transfer of microbial biomass to the mid and bottom canopy where reduced physical impact of rainfall [12], more favourable microclimatic conditions, and higher nutrient availability might allow a denser colonization with more efficient protection against detachment. Despite the strong effect on vertical phyllosphere abundance and diversity patterns, transport-related mechanisms did not result in consistent canopy position dependent community patterns across all tree species and phenological stages, as canopy position only explained a maximum of 6% of total community variation. The fact that canopy position contributed 15% of community variation in September 2017 at the same study site [11] may point to an important role of the specific weather conditions of a given year for vertical patterns of the phyllosphere microbiome. Along with affecting within-canopy transport dynamics, weather conditions during the growing season may influence the time point of leaf senescence onset and its progression [66]. As the extent of senescence may, in turn, differ between the top, mid and bottom of the canopy [67], senescence-induced vertical gradients of phyllosphere microhabitat conditions may vary from year to year, resulting in a different extent of vertical community differentiation in autumn.

In addition, the vertical distribution of individual bacterial taxa appeared to be strongly influenced by potential airborne import and attachment and detachment patterns during exchange between throughfall and phyllosphere. Taxa typically associated with cloud water or atmospheric inputs such as *Massilia* [68–70] or *Pseudomonas* [70] were abundant in rainwater above the canopy and in throughfall but exhibited a strong tendency to preferentially occur in the phyllosphere, pointing to strong adhesion mechanisms. In fact, 10–40% of taxa typically occurring in the phyllosphere are known to form biofilms for attachment [71], including the genera *Hymenobacter* sp., *Massilia* sp., and *Pseudomonas* sp. which dominated the phyllosphere communities in this study. A stronger tendency for an attached lifestyle of *Massilia* sp. in association with oak, *Halotalea* sp. in association with ash, and unclassified *Myxococcaceae* in association with linden, further pointed to a central role of the host tree in selectively favoring attachment versus wash-off of individual taxa.

Similar to the phyllosphere communities, we observed a strong seasonal imprint on throughfall bacterial community composition. Throughfall collected in March during the leafless stage of the canopy harboured the most distinct bacterial communities, suggesting that microbial exchange between throughfall and phyllosphere after foliage emerged had a strong converging effect on the transported communities.

## Conclusions

Our findings revealed a strong impact of phenological stages on bacterial communities and their assembly in the phyllosphere of temperate forests, exceeding tree species-specific or canopy position dependent effects. Yet, an increasing influence of tree species identity towards mid and late phenological stages, along with increased functional redundancy, suggested a transition from phyllosphere communities which are mostly controlled by random colonization events and dispersal limitation in spring towards communities which are increasingly subject to tree-species specific controls. Our unique experimental setup further identified throughfall as a key mechanism importing and transporting bacteria through the tree crowns with taxon-specific mobilization patterns, which likely underlies the observed maxima of diversity and phyllosphere bacterial abundance at the bottom of the canopy. Our work underscores the need to integrate phenological aspects as well as the whole vertical extension of tree canopies to understand phyllosphere microbiome dynamics in temperate forests.

## Supplementary Information

Below is the link to the electronic supplementary material.

Supplementary Material 1: Additional file 1.xlsx The installation heights of throughfall collectors at crane top and different canopy positions (Top, middle and bottom) and the maximum height of the tree individuals.

Supplementary Material 2: Additional file 2.xlsx Linear mixed-effects model (LMM) results for (1) phyllosphere bacterial abundance, (2) throughfall bacterial abundance, (3) throughfall bacterial flux, (4) phyllosphere Shannon diversity, (5) throughfall Shannon diversity, (6) phyllosphere Beijerinckiaceae, (7) phyllosphere Erwiniaceae, (8) throughfall Beijerinckiaceae, and (9) throughfall Erwiniaceae. Values represent model estimates, standard errors (SE), degrees of freedom (df), test statistics (t), and P-values.

Supplementary Material 3: Additional file 3.xlsx The mean estimated cell abundances of phyllosphere (cells/gram leaf dry weight) and throughfall (cells/liter of throughfall) at canopy position level. The estimated cell abundances were calculated based on the following calculation, [relative abundance/average 16S rRNA operon number (at family level)] X 16S rRNA gene abundance data determined by qPCR. Briefly, the relative abundance values were divided by the family level mean 16S rRNA operon numbers (https://rrndb.umms.med.umich.edu/), then multiplied with qPCR abundance data.

Supplementary Material 4: Additional file 4.xlsx The complete list of differentially abundant amplicon sequence variants (ASV) at genus level in the phyllosphere and throughfall samples across phenological stages (P<0.05 and FDR<0.05) based on Linear discriminant analysis Effect Size (LEfSe) analysis.

Supplementary Material 5: Additional file 5.xlsx Successional and vertical trends of phyllosphere attachment/throughfall wash–off patterns of amplicon sequence variants. The relative abundances of common ASVs between groups (crane top vs throughfall or phyllosphere vs throughfall) were analyzed at a) successional (May, July and October) and b) vertical levels (crane top, top, middle and bottom canopy positions). LDA scores were calculated using LEfSe (P<0.05 and FDR<0.05).

Supplementary Material 6: Additional file 6.xlsx Bin contributions to cumulative community assembly processes across tree species, canopy positions, and phenological stages. This data file shows both bin and group-level patterns of relative importance of different community assembly processes. Initially, bin-wise abundance-weighted contribution to ecological assembly process at group level (defined by tree species, sampling month, and canopy position with three replicates per group) (e.g., Oak_Top_May) was calculated. Abundance-weighted bin contributions were obtained by multiplying bin relative abundance (BinRA) by the corresponding bin-level process importance, and these values were aggregated across bins to estimate cumulative process relative importance at the group level.

Supplementary Material 7: Additional file 7.xlsx Bin-level ecological assembly processes inferred using iCAMP. Bin-wise community assembly processes are shown across tree species (Oak, Ash, Linden), sampling months (May, July, October), and canopy positions (Top, Middle and Bottom), with three replicates per group. Columns represent individual sample groups, and rows correspond to phylogenetic bins. The dominant process (heterogeneous selection, homogeneous selection, dispersal limitation, homogenizing dispersal, and drift) within each bin is identified as the process with the highest relative importance and is shown together with its associated P-value, indicating statistical support at the bin level.

Supplementary Material 8: Additional file 8.xlsx The comparative analysis of KEGG pathway enrichment across phenological stages (Pairwise seasonal comparisons: May vs. July and July vs. October) are shown across three host tree species (Oak, Ash, and Linden). Pathways are annotated according to KEGG functional hierarchy levels L2 and L3. Statistical significance of differences between sampling periods (May vs. July and July vs. October) was evaluated separately for each tree species using the Kruskal–Wallis test followed by Dunn’s post hoc pairwise comparisons. Significance is reported as P-values and indicated as TRUE or FALSE based on a threshold of P<0.05, as implemented in the stats package in R.

Supplementary Material 9

Supplementary Material 10

## Data Availability

The amplicon sequencing datasets generated in this study have been deposited under the BioProject IDs PRJEB109949 and PRJEB109950 in the European Nucleotide Archive (ENA). The codes used in this study have been deposited in Zenodo and are available at https://zenodo.org/records/21373404 (DOI: 10.5281/zenodo.21373404). Results of quantitative PCR, PICRUSt analysis and community assembly analysis are provided as Supplementary Material. The datasets supporting the conclusions of this article are included within the article and its additional files.

## References

[1] Morris CE, Kinkel L. Fifty years of phyllosphere microbiology: significant contributions to research in related fields. In: Phyllosphere microbiology. 2002;22:365–75.

[2] Laforest-Lapointe I, Whitaker BK. Decrypting the phyllosphere microbiota: progress and challenges. Am J Bot. 2019;106:171–3. 10.1002/ajb2.1229 30726571

[3] Schäfer M, Vogel CM, Bortfeld-Miller M, Mittelviefhaus M, Vorholt JA. Mapping phyllosphere microbiota interactions in planta to establish genotype–phenotype relationships. Nat Microbiol. 2022;7:856–67. 10.1038/s41564-022-01132-w PMC7612798 35637327

[4] Carrell AA, Frank AC. Pinus flexilis and Piceae engelmannii share a simple and consistent needle endophyte microbiota with a potential role in nitrogen fixation. Front Microbiol. 2014;5:333. 10.3389/fmicb.2014.00333 PMC4082182 25071746

[5] Iguchi H, Yurimoto H, Sakai Y. Interactions of Methylotrophs with Plants and Other Heterotrophic Bacteria. Microorganisms. 2015;3:137–51. 10.3390/microorganisms3020137 PMC5023238 27682083

[6] Venkatachalam S, Ranjan K, Prasanna R, Ramakrishnan B, Thapa S, Kanchan A. Diversity and functional traits of culturable microbiome members, including cyanobacteria in the rice phyllosphere. Papen H, editor. Plant Biol. 2016;18:627–37. 10.1111/plb.12441 26849835

[7] De Mandal S, Jeon J. Phyllosphere Microbiome in Plant Health and Disease. Plants. 2023;12:3481. 10.3390/plants12193481 PMC10575124 37836221

[8] Ginnan NA, De Anda NI, Campos Freitas Vieira F, Rolshausen PE, Roper MC. Microbial turnover and dispersal events occur in synchrony with plant phenology in the perennial evergreen tree crop Citrus sinensis. MBio. 2022;13. 10.1128/mbio.00343-22 PMC9239260 35642946

[9] Al Ashhab A, Meshner S, Alexander-Shani R, Dimerets H, Brandwein M, Bar-Lavan Y, et al. Temporal and spatial changes in phyllosphere microbiome of Acacia trees growing in arid environments. Front Microbiol. 2021;12:656269. 10.3389/fmicb.2021.656269 PMC8312645 34322096

[10] Laforest-Lapointe I, Messier C, Kembel SW. Host species identity, site and time drive temperate tree phyllosphere bacterial community structure. Microbiome. 2016;4:27. 10.1186/s40168-016-0174-1 PMC4912770 27316353

[11] Herrmann M, Geesink P, Richter R, Küsel K. Canopy Position Has a Stronger Effect than Tree Species Identity on Phyllosphere Bacterial Diversity in a Floodplain Hardwood Forest. Microb Ecol. 2021;81:157–68. 10.1007/s00248-020-01565-y PMC7794210 32761502

[12] Stone BWG, Jackson CR. Canopy position is a stronger determinant of bacterial community composition and diversity than environmental disturbance in the phyllosphere. FEMS Microbiol Ecol. 2019;95:fiz032. 10.1093/femsec/fiz032 30860575

[13] Tukey HB. The Leaching of Substances from Plants. Annu Rev Plant Physiol. 1970;21:305–24.

[14] Van Stan JT, Levia DF, Inamdar SP, Lepori-Bui M, Mitchell MJ. The effects of phenoseason and storm characteristics on throughfall solute washoff and leaching dynamics from a temperate deciduous forest canopy. Sci Total Environ. 2012;430:48–58. 10.1016/j.scitotenv.2012.04.060 22621811

[15] Feeny P. Seasonal Changes in Oak Leaf Tannins and Nutrients as a Cause of Spring Feeding by Winter Moth Caterpillars. Ecology. 1970;51:565–81.

[16] Mediavilla S, Escudero A. Relative growth rate of leaf biomass and leaf nitrogen content in several mediterranean woody species. Plant Ecol. 2003;168:321–32.

[17] Sancho-Knapik D, Martín-Sánchez R, Alonso-Forn D, Peguero-Pina JJ, Ferrio JP, Gil-Pelegrín E. Trade-offs among leaf toughness, constitutive chemical defense, and growth rates in oaks are influenced by the level of leaf mass per area. Ann Sci. 2023;80:39.

[18] Chen D, Yang J, Wang S, Lan S, Wang Y, Liu Z-J, et al. Comparative analysis of community composition and network structure between phyllosphere endophytic and epiphytic fungal communities of Mussaenda pubescens. Hockett KL, editor. Microbiol Spectr. 2025;13. 10.1128/spectrum.01019-24 PMC11705847 39625383

[19] Deng N, Liu C, Tian Y, Song Q, Niu Y, Ma F. Assembly processes of rhizosphere and phyllosphere bacterial communities in constructed wetlands created via transformation of rice paddies. Front Microbiol. 2024;15:1337435. 10.3389/fmicb.2024.1337435 PMC10913029 38444812

[20] Li T, Gao J. Attribution of dispersal limitation can better explain the assembly patterns of plant microbiota. Front Plant Sci. 2023;14: article 1168760. 10.3389/fpls.2023.1168760 PMC10628812 37941678

[21] Chase JM, Myers JA. Disentangling the importance of ecological niches from stochastic processes across scales. Philos Trans R Soc B Biol Sci. 2011;366:2351–63. 10.1098/rstb.2011.0063 PMC3130433 21768151

[22] Ponette-González AG, Van Stan IIJT, Magyar D. Things Seen and Unseen in Throughfall and Stemflow. Precip Partitioning by Veg. Cham: Springer International Publishing; 2020. pp. 71–88.

[23] Zhou S-Y-D, Li H, Giles M, Neilson R, Yang X, Su J. Microbial flow within an air-Phyllosphere-soil Continuum. Front Microbiol. 2021;11: article 615481. 10.3389/fmicb.2020.615481 PMC7873851 33584580

[24] Levia DF, Keim RF, Carlyle-Moses DE, Frost EE. Throughfall and stemflow in wooded ecosystems. 2011. pp. 425-443.

[25] Lusk MG. Throughfall as an understudied biogeochemical subsidy of nutrients and carbon in the urban water cycle: perspective and a research agenda. Discov Water. 2024;4:124.

[26] Bittar TB, Pound P, Whitetree A, Moore LD, Van Stan JT. Estimation of Throughfall and Stemflow Bacterial Flux in a Subtropical Oak-Cedar Forest. Geophys Res Lett. 2018;45:1410–8.

[27] Hassan A, Frank J. Influence of surfactant hydrophobicity on the detachment of Escherichia coli O157:H7 from lettuce. Int J Food Microbiol. 2003;87:145–52. 10.1016/s0168-1605(03)00062-x 12927717

[28] Pantanella F, Berlutti F, Passariello C, Sarli S, Morea C, Schippa S. Violacein and biofilm production in Janthinobacterium lividum. J Appl Microbiol. 2006;102:992–9. 10.1111/j.1365-2672.2006.03155.x 17381742

[29] Lyautey E, Jackson CR, Cayrou J, Rols J-L, Garabétian F. Bacterial Community Succession in Natural River Biofilm Assemblages. Microb Ecol. 2005;50:589–601. 10.1007/s00248-005-5032-9 16341639

[30] Teachey M, Pound P, Ottesen E, Van Stan J. Bacterial Community Composition of Throughfall and Stemflow. Front Glob Chang. 2018;1:7.

[31] Krüger M, Potthast K, Michalzik B, Tischer A, Herrmann M. Rainwater-driven transport of matter and microbes from phyllosphere to soil in a temperate beech forest. Sci Total Environ. 2025;1006:180910. 10.1016/j.scitotenv.2025.180910 41237615

[32] Jansen E. Das Naturschutzgebiet Burgaue. Leipzig: Staatliches Umweltfachamt; 1999.

[33] Richter R, Ballasus H, Engelmann RA, Zielhofer C, Sanaei A, Wirth C. Tree species matter for forest microclimate regulation during the drought year 2018: disentangling environmental drivers and biotic drivers. Sci Rep. 2022;12:17559. 10.1038/s41598-022-22582-6 PMC9584904 36266469

[34] Patzak R, Richter R, Engelmann RA, Wirth C. Tree crowns as meeting points of diversity generating mechanisms – a test with epiphytic lichens in a temperate forest. 2020, preprint on bioRxiv.

[35] Richter R, Reu B, Wirth C, Doktor D, Vohland M. The use of airborne hyperspectral data for tree species classification in a species-rich Central European forest area. Int J Appl Earth Obs Geoinf. 2016;52:464–74.

[36] Klindworth A, Pruesse E, Schweer T, Peplies J, Quast C, Horn M, et al. Evaluation of general 16S ribosomal RNA gene PCR primers for classical and next-generation sequencing-based diversity studies. Nucleic Acids Res. 2013;41:e1. 10.1093/nar/gks808 PMC3592464 22933715

[37] Chelius MK, Triplett EW. The diversity of archaea and bacteria in association with the roots of Zea mays L. Microb Ecol. 2001;41:252–63. [cited 2014 Jan 28]. 10.1007/s002480000087 11391463

[38] Krüger M, Potthast K, Michalzik B, Tischer A, Küsel K, Deckner FFK, et al. Drought and rewetting events enhance nitrate leaching and seepage-mediated translocation of microbes from beech forest soils. Soil Biol Biochem. 2021;154:108153.

[39] Callahan BJ, McMurdie PJ, Rosen MJ, Han AW, Johnson AJA, Holmes SP. DADA2: High-resolution sample inference from Illumina amplicon data. Nat Methods. 2016;13:581–3. 10.1038/nmeth.3869 PMC4927377 27214047

[40] Jiao S, Lu Y. Soil pH and temperature regulate assembly processes of abundant and rare bacterial communities in agricultural ecosystems. Environ Microbiol. 2020;22:1052–65. 10.1111/1462-2920.14815 31599105

[41] Douglas GM, Maffei VJ, Zaneveld JR, Yurgel SN, Brown JR, Taylor CM, et al. PICRUSt2 for prediction of metagenome functions. Nat Biotechnol. 2020;38:685–8. 10.1038/s41587-020-0548-6 PMC7365738 32483366

[42] Ning D, Yuan M, Wu L, Zhang Y, Guo X, Zhou X, et al. A quantitative framework reveals ecological drivers of grassland microbial community assembly in response to warming. Nat Commun. 2020;11:4717. 10.1038/s41467-020-18560-z PMC7501310 32948774

[43] Stegen JC, Lin X, Fredrickson JK, Chen X, Kennedy DW, Murray CJ, et al. Quantifying community assembly processes and identifying features that impose them. ISME J. 2013;7:2069–79. 10.1038/ismej.2013.93 PMC3806266 23739053

[44] Oksanen J, Blanchet FG, Friendly M, Kindt R, Legendre P, McGlinn D, et al. vegan community ecology package version 2.5-7 November 2020. 2020.

[45] Bao L, Gu L, Sun B, Cai W, Zhang S, Zhuang G, et al. Seasonal variation of epiphytic bacteria in the phyllosphere of Gingko biloba, Pinus bungeana and Sabina chinensis. FEMS Microbiol Ecol. 2020;96:017. 10.1093/femsec/fiaa017 32005997

[46] Wang J, Shi X, Lucas-Borja ME, Guo Q, Wang L, Huang Z. Contribution of tree species to the co-occurrence network of the leaf phyllosphere and soil bacterial community in the subtropical forests. J Environ Manage. 2023;343:118274. 10.1016/j.jenvman.2023.118274 37247543

[47] Laforest-Lapointe I, Messier C, Kembel SW. Tree leaf bacterial community structure and diversity differ along a gradient of urban intensity. mSystems. 2017;2. 10.1128/mSystems.00087-17 PMC5715107 29238751

[48] Ekholm A, Tack AJM, Pulkkinen P, Roslin T. Host plant phenology, insect outbreaks and herbivore communities – The importance of timing. Zytynska S, editor. J Anim Ecol. 2020;89:829–41. 10.1111/1365-2656.13151 31769502

[49] Wong CYS, D’Odorico P, Arain MA, Ensminger I. Tracking the phenology of photosynthesis using carotenoid-sensitive and near‐infrared reflectance vegetation indices in a temperate evergreen and mixed deciduous forest. New Phytol. 2020;226:1682–95. 10.1111/nph.16479 32039477

[50] Vermeuel MP, Novak GA, Kilgour DB, Claflin MS, Lerner BM, Trowbridge AM, et al. Observations of biogenic volatile organic compounds over a mixed temperate forest during the summer to autumn transition. Atmos Chem Phys. 2023;23:4123–48.

[51] Hagen-Thorn A, Varnagiryte I, Nihlgård B, Armolaitis K. Autumn nutrient resorption and losses in four deciduous forest tree species. Ecol Manage. 2006;228:33–9.

[52] Cullen NP, Fetters AM, Ashman T-L. Integrating microbes into pollination. Curr Opin Insect Sci. 2021;44:48–54. 10.1016/j.cois.2020.11.002 33248285

[53] Holopainen JK, Leinonen R, Blande JD, Sorvari J. Aphid honeydew in ecosystems and the environment. Arthropod-Plant Interact. 2026;20:53.

[54] Hoffland E, Jeger MJ, Van Beusichem ML. Effect of nitrogen supply rate on disease resistance in tomato depends on the pathogen. Plant Soil. 2000;218:239–47.

[55] Posfai A, Taillefumier T, Wingreen NS. Metabolic Trade-Offs Promote Diversity in a Model Ecosystem. Phys Rev Lett. 2017;118:028103. 10.1103/PhysRevLett.118.028103 PMC5743855 28128613

[56] Chen Y, Poland TM. Interactive Influence of Leaf Age, Light Intensity, and Girdling on Green Ash Foliar Chemistry and Emerald Ash Borer Development. J Chem Ecol. 2009;35:806–15. 10.1007/s10886-009-9661-1 19568811

[57] Tamas I, Smirnova AV, He Z, Dunfield PF. The (d)evolution of methanotrophy in the Beijerinckiaceae —a comparative genomics analysis. ISME J. 2014;8:369–82. 10.1038/ismej.2013.145 PMC3906808 23985741

[58] Maignien L, DeForce EA, Chafee ME, Eren AM, Simmons SL. Ecological succession and stochastic variation in the assembly of Arabidopsis thaliana phyllosphere communities. mBio. 2014;5. 10.1128/mBio.00682-13 PMC3903271 24449749

[59] Schlechter RO, Kear EJ, Bernach M, Remus DM, Remus-Emsermann MNP. Metabolic resource overlap impacts competition among phyllosphere bacteria. ISME J. 2023;17:1445–54. 10.1038/s41396-023-01459-0 PMC10432529 37355740

[60] Ai W, Liu H, Wang Y, Wang Y, Wei J, Zhang X, et al. Identification of Functional Brassinosteroid Receptor Genes in Oaks and Functional Analysis of QmBRI1. Int J Mol Sci. 2023;24:16405. 10.3390/ijms242216405 PMC10671120 38003597

[61] Bertić M, Orgel F, Gschwendtner S, Schloter M, Moritz F, Schmitt-Kopplin P, et al. European oak metabolites shape digestion and fitness of the herbivore Tortrix viridana. Funct Ecol. 2023;37:1476–91.

[62] Zhou J, Ning D. Stochastic community assembly: does it matter in microbial ecology? Microbiol Mol Biol Rev. 2017;81:e00002-17. 10.1128/MMBR.00002-17 PMC5706748 29021219

[63] Ren Y, Xun W, Yan H, Ma A, Xiong W, Shen Q, et al. Functional compensation dominates the assembly of plant rhizospheric bacterial community. Soil Biol Biochem. 2020;150:107968.

[64] Li Q, Long Z, Wang H, Zhang G. Functions of constructed wetland animals in water environment protection – A critical review. Sci Total Environ. 2021;760:144038. 10.1016/j.scitotenv.2020.144038 33348153

[65] Duan Y, Siegenthaler A, Skidmore AK, Chariton AA, Laros I, Rousseau M, et al. Forest top canopy bacterial communities are influenced by elevation and host tree traits. Environ Microbiome. 2024;19:21. 10.1186/s40793-024-00565-6 PMC10998314 38581032

[66] Zohner CM, Mirzagholi L, Renner SS, Mo L, Rebindaine D, Bucher R, Palous D, Vitasse Y, Fu YH, Stocker BD, Crowther TW. Effect of climate warming on the timing of autumn leave senescence reverses after the summer solstice. Science. 2023;381:eadf5098. 10.1126/science.adf5098 37410847

[67] Zahnd C, Arend M, Kahmen A, Hoch G. (2023). Microclimatic gradients cause phenological variations within temperate three canopies in autumn but not in spring. Agric. For. Meteorol. 2023;331:109340.

[68] Zhang Y, Du R, Chen H, Du P, Zhang S, Ren W. Different characteristics of microbial diversity and special functional microbes in rainwater and topsoil before and after 2019 new coronavirus epidemic in Inner Mongolia Grassland. Sci Total Environ. 2022;809:151088. 10.1016/j.scitotenv.2021.151088 PMC8527739 34687707

[69] Li X, Chen H, Yao M. Microbial emission levels and diversities from different land use types. Environ Int. 2020;143:105988. 10.1016/j.envint.2020.105988 32717647

[70] Woo C, Yamamoto N. Falling bacterial communities from the atmosphere. Environ Microbiome. 2020;15:22. 10.1186/s40793-020-00369-4 PMC8066439 33902752

[71] Morris CE, Monier J-M, Jacques M-A. A Technique To Quantify the Population Size and Composition of the Biofilm Component in Communities of Bacteria in the Phyllosphere. Appl Environ Microbiol. 1998;64:4789–95. 10.1128/aem.64.12.4789-4795.1998 PMC90923 9835563

