# Supplementary material for: Traversing the canopy: phenology-driven changes and within-canopy transport shape the phyllosphere microbiome in a temperate floodplain hardwood forest
Source: Environ Microbiome. 2026 Nov 12;21:135. doi: 10.1186/s40793-026-00984-7 (PMC13769150; doi:10.1186/s40793-026-00984-7)
Supplement: Supplementary file 2 [file 40793_2026_984_MOESM9_ESM.docx]

**Supplementary tables:**

**Table S1.** Metadata (Latitude, Longitude, Diameter at Breast Height (DBH), Altitude) of the tree individuals sampled at the Leipzig Canopy Crane facility, Germany.

| **Tree individual** | **Tree species** | **Latitude** | **Longitude** | **DBH (cm)** | **Altitude (m NN)** | **Predicted age (y)** | **Minimum age (y)** |
| --- | --- | --- | --- | --- | --- | --- | --- |
| ID129 | *Quercus robur* | 51.3651972 | 12.3089685 | 136.7 | 102 | 370 | - |
| ID131 | *Quercus robur* | 51.3651568 | 12.3088270 | 100.6 | 102 | - | - |
| ID733 | *Quercus robur* | 51.3651312 | 12.3098006 | 55.5 | 102 | 102 | - |
| ID232 | *Fraxinus excelsior* | 51.3656189 | 12.3090301 | 68.7 | 102 | - | - |
| ID435 | *Fraxinus excelsior* | 51.3663795 | 12.3092601 | 81.5 | 102 | - | - |
| ID770 | *Fraxinus excelsior* | 51.3650268 | 12.3095312 | 84.0 | 102 | - | - |
| ID439 | *Tilia cordata* | 51.3662962 | 12.3091490 | 76.8 | 102 | - | - |
| ID455 | *Tilia cordata* | 51.3663526 | 12.3096566 | 71 | 102 | 205 | 129 |
| ID517 | *Tilia cordata* | 51.3662235 | 12.3098308 | 81 | 102 | 235 | 148 |
|  |  |  |  |  |  |  |  |

**Table S2.** PERMANOVA analysis for the effects of **month, host species, position, and their interactions on bacterial communities** of a) phyllosphere, b) throughfall bacterial communities c) month-wise for phyllosphere. R²: proportion of variance explained by each factor. F: F-statistic indicating the strength of the effect. Pr(>F): *P*-value, with significance levels indicated as: *** *P*<0.001, ** *P*<0.01, * *P*<0.05. Unexplained: proportion of variance not accounted for by the model. Tree individual was included as a **fixed effect** in the PERMANOVA to assess variation attributable to individual trees.

a)

|  | **R²** | **F** | **Pr(>F)** |
| --- | --- | --- | --- |
| Month | 22.598 | 34.3070 | 0.001 *** |
| Host | 12.794 | 19.4232 | 0.001 *** |
| Position | 2.153 | 3.2693 | 0.001 *** |
| Month*Host | 6.709 | 5.0925 | 0.001 *** |
| Host*Position | 1.778 | 1.3499 | 0.039 * |
| Month*Position | 1.931 | 1.4661 | 0.007 ** |
| Unexplained | 52.037 |  |  |

b)

|  | R2 | F | Pr(>F) |
| --- | --- | --- | --- |
| Month | 19.984 | 30.4734 | 0.001*** |
| Host | 4.384 | 10.027 | 0.001*** |
| Position | 2.221 | 5.0794 | 0.001*** |
| Month:Host | 4.501 | 3.4316 | 0.001*** |
| Host:Position | 1.483 | 1.6965 | 0.001** |
| Month:Position | 2.942 | 2.2432 | 0.001*** |
| Residual | 64.485 |  |  |

c)

|  | **R^2^** | **F** | **Pr(>F)** |
| --- | --- | --- | --- |
| **May** |  |  |  |
| Host | 19.46 | 7.2071 | 0.003 *** |
| Position | 4.89 | 1.8129 | 0.003 *** |
| **July** |  |  |  |
| Host | 27.36 | 11.439 | 0.001 *** |
| Position | 5.66 | 2.369 | 0.001 *** |
| **October** |  |  |  |
| Host | 32.86 | 13.414 | 0.001 *** |
| Position | 5.89 | 2.404 | 0.004 ** |

**Table S3.** **Linear mixed-effects model results for estimated bacterial abundances, bacterial flux, Shannon’s diversity index (H’), and selected bacterial family abundances in the phyllosphere and throughfall.** Models included tree species, phenological stage, canopy position, and their two-way interactions as fixed effects, with tree individual included as a random effect to account for repeated sampling of individual trees. Values represent P-values from Type III tests of fixed effects. **Bold values indicate statistically significant effects (**P<0.05**).** “Phenological stage” represents the early, mid, and late sampling stages (May, July, and October).

| **Response** | **Tree species** | **Phenological stage** | **Canopy position** | **Tree species × Phenological stage** | **Tree species × Canopy position** | **Phenological stage × Canopy position** |
| --- | --- | --- | --- | --- | --- | --- |
| Phyllosphere bacterial abundance | 0.346 | 0.157 | **0.0065** | **<0.001** | 0.639 | 0.597 |
| Throughfall bacterial abundance | 0.419 | **<0.001** | **0.0031** | **<0.001** | 0.118 | **<0.001** |
| Throughfall bacterial flux | 0.270 | **<0.001** | 0.061 | **<0.001** | 0.197 | **0.0013** |
| Phyllosphere Shannon | 0.0715 | **<0.001** | **<0.001** | 0.256 | **0.0099** | 0.746 |
| Throughfall Shannon | 0.978 | **<0.001** | **<0.001** | 0.283 | 0.401 | 0.070 |
| Phyllosphere *Beijerinckiaceae* | 0.642 | **0.0039** | **0.0101** | **0.0032** | 0.160 | 0.795 |
| Phyllosphere *Erwiniaceae* | 0.335 | **0.0423** | 0.878 | **<0.001** | 0.723 | 0.317 |
| Throughfall *Beijerinckiaceae* | 0.244 | **<0.001** | **<0.001** | 0.309 | 0.327 | **<0.001** |
| Throughfall *Erwiniaceae* | 0.928 | **<0.001** | 0.589 | **0.00125** | 0.801 | **0.00142** |

**Table S4.** a) The relative percentage of successional and newly introduced ASVs across the phenological succession. July represents the May to July succession, while October represents the July to October succession. b) Relative proportions of ASV categories (abundant, intermediate, rare) across phenological stages for (a) successional and (b) newly introduced ASVs.

|  | Successional | New intro |
| --- | --- | --- |
| Oak_Top_July | 65.877 | 34.123 |
| Oak_Top_October | 91.623 | 8.377 |
| Oak_Middle_July | 75.374 | 24.626 |
| Oak_Middle_October | 87.900 | 12.100 |
| Oak_Bottom_July | 75.028 | 24.972 |
| Oak_Bottom_October | 89.831 | 10.169 |
| Ash_Top_July | 55.438 | 44.562 |
| Ash_Top_October | 88.413 | 11.587 |
| Ash_Middle_July | 49.545 | 50.455 |
| Ash_Middle_October | 89.240 | 10.760 |
| Ash_Bottom_July | 76.032 | 23.968 |
| Ash_Bottom_October | 91.758 | 8.242 |
| Linden_Top_July | 76.691 | 23.309 |
| Linden_Top_October | 86.362 | 13.638 |
| Linden_Middle_July_ | 73.572 | 26.428 |
| Linden_Middle_October | 89.362 | 10.638 |
| Linden_Bottom_July | 84.197 | 15.803 |
| Linden_Bottom_October | 87.192 | 12.808 |

| Successional ASV | Abundant | Intermediate | Rare |
| --- | --- | --- | --- |
| Oak_Top_July | 43.382 | 40.074 | 16.544 |
| Oak_Top_October | 22.959 | 35.034 | 42.007 |
| Oak_Middle_July | 40.286 | 41.429 | 18.286 |
| Oak_Middle_October | 25.950 | 49.421 | 24.628 |
| Oak_Bottom_July | 36.675 | 45.910 | 17.414 |
| Oak_Bottom_October | 31.507 | 42.466 | 26.027 |
| Ash_Top_July | 17.327 | 34.158 | 48.515 |
| Ash_Top_October | 13.924 | 47.152 | 38.924 |
| Ash_Middle_July | 2.745 | 19.216 | 78.039 |
| Ash_Middle_October | 11.278 | 40.226 | 48.496 |
| Ash_Bottom_July | 23.383 | 36.816 | 39.801 |
| Ash_Bottom_October | 16.271 | 41.356 | 42.373 |
| Linden_Top_July | 48.661 | 27.679 | 23.661 |
| Linden_Top_October | 41.261 | 47.851 | 10.888 |
| Linden_Middle_July | 43.581 | 41.216 | 15.203 |
| Linden_Middle_October | 30.909 | 42.045 | 27.045 |
| Linden_Bottom_July | 46.246 | 39.339 | 14.414 |
| Linden_Bottom_October | 30.538 | 46.452 | 23.011 |

| Newly introduced ASVs | Abundant | Intermediate | Rare |
| --- | --- | --- | --- |
| Oak_Top_July | 10.047 | 40.973 | 48.980 |
| Oak_Top_October | 3.247 | 30.519 | 66.234 |
| Oak_Middle_July | 8.775 | 47.532 | 43.693 |
| Oak_Middle_October | 2.811 | 35.141 | 62.048 |
| Oak_Bottom_July | 7.078 | 43.739 | 49.183 |
| Oak_Bottom_October | 4.814 | 40.481 | 54.705 |
| Ash_Top_July | 3.689 | 18.577 | 77.734 |
| Ash_Top_October | 0.939 | 19.014 | 80.047 |
| Ash_Middle_July | 0.996 | 7.255 | 91.750 |
| Ash_Middle_October | 0.334 | 18.060 | 81.605 |
| Ash_Bottom_July | 4.839 | 31.452 | 63.710 |
| Ash_Bottom_October | 1.180 | 20.059 | 78.761 |
| Linden_Top_July | 11.873 | 41.472 | 46.656 |
| Linden_Top_October | 9.920 | 54.424 | 35.657 |
| Linden_Middle_July | 10.766 | 46.770 | 42.464 |
| Linden_Middle_October | 3.885 | 37.832 | 58.282 |
| Linden_Bottom_July | 13.987 | 45.511 | 40.501 |
| Linden_Bottom_October | 3.735 | 36.163 | 60.102 |

**Table S5.** Amplicon sequence variants (ASVs) detected only during a) July or b) October at tree species and canopy position levels

a)

| **Host** | **ASV** | **Family** | **Genus** |
| --- | --- | --- | --- |
| Oak_Top | ASV89 | *Microbacteriaceae* | *Rathayibacter* |
| Oak_Top | ASV47 | *Nocardiaceae* | *Rhodococcus* |
| Oak_Top | ASV50 | *Sphingobacteriaceae* | *Pedobacter* |
| Oak_Top | ASV45 | *Oxalobacteraceae* | *Massilia* |
| Oak_Middle | ASV47 | *Nocardiaceae* | *Rhodococcus* |
| Oak_Middle | ASV49 | *Sphingobacteriaceae* | *Pedobacter* |
| Oak_Middle | ASV25 | *Sphingobacteriaceae* | *Pedobacter* |
| Oak_Bottom | ASV50 | *Sphingobacteriaceae* | *Pedobacter* |
| Ash_Top | ASV89 | *Microbacteriaceae* | *Rathayibacter* |
| Ash_Top | ASV47 | *Nocardiaceae* | *Rhodococcus* |
| Ash_Top | ASV49 | *Sphingobacteriaceae* | *Pedobacter* |
| Ash_Top | ASV94 | *Oxalobacteraceae* | *Massilia* |
| Ash_Top | ASV87 | *Comamonadaceae* | *Variovorax* |
| Ash_Middle | ASV71 | *Rhizobiaceae* | *Allorhizobium-Neorhizobium-Pararhizobium-Rhizobium* |
| Ash_Middle | ASV89 | *Microbacteriaceae* | *Rathayibacter* |
| Ash_Middle | ASV47 | *Nocardiaceae* | *Rhodococcus* |
| Ash_Middle | ASV40 | *Rhizobiaceae* | *Ensifer* |
| Ash_Middle | ASV28 | *Comamonadaceae* | *Variovorax* |
| Ash_Middle | ASV95 | *Beijerinckiaceae* | |
| Ash_Middle | ASV84 | *Spirosomaceae* | *Spirosoma* |
| Ash_Middle | ASV46 | *Xanthobacteraceae* | *Rhodopseudomonas* |

| **Host** | **ASV** | **Family** | **Genus** |
| --- | --- | --- | --- |
| Ash_Bottom | ASV81 | *Morganellaceae* | *Buchnera* |
| Ash_Bottom | ASV52 | *Oxalobacteraceae* | *Massilia* |
| Ash_Bottom | ASV47 | *Nocardiaceae* | *Rhodococcus* |
| Ash_Bottom | ASV65 | *Oxalobacteraceae* | *Massilia* |
| Ash_Bottom | ASV87 | *Comamonadaceae* | *Variovorax* |
| Linden_Top | ASV77 | *Hymenobacteraceae* | *Hymenobacter* |
| Linden_Top | ASV100 | *Oxalobacteraceae* | *Massilia* |
| Linden_Top | ASV42 | *Microbacteriaceae* | |
| Linden_Top | ASV89 | *Microbacteriaceae* | *Rathayibacter* |
| Linden_Top | ASV96 | *Yersiniaceae* | *Serratia* |
| Linden_Top | ASV33 | *Yersiniaceae* | *Serratia* |
| Linden_Top | ASV39 | *Oxalobacteraceae* | *Massilia* |
| Linden_Middle | ASV81 | *Morganellaceae* | *Buchnera* |
| Linden_Middle | ASV90 | *Oxalobacteraceae* | *Massilia* |
| Linden_Middle | ASV100 | *Oxalobacteraceae* | *Massilia* |
| Linden_Middle | ASV40 | *Rhizobiaceae* | *Ensifer* |
| Linden_Middle | ASV62 | *Oxalobacteraceae* | *Massilia* |
| Linden_Middle | ASV28 | *Comamonadaceae* | *Variovorax* |
| Linden_Middle | ASV84 | *Spirosomaceae* | *Spirosoma* |
| Linden_Middle | ASV46 | *Xanthobacteraceae* | *Rhodopseudomonas* |
| Linden_Middle | ASV89 | *Microbacteriaceae* | *Rathayibacter* |
| Linden_Middle | ASV39 | *Oxalobacteraceae* | *Massilia* |
| Linden_Middle | ASV72 | *Oxalobacteraceae* | *Duganella* |
| Linden_Middle | ASV60 | *Pseudomonadaceae* | *Pseudomonas* |
| Linden_Middle | ASV59 | *Oxalobacteraceae* | *Janthinobacterium* |
| Linden_Middle | ASV75 | *Spirosomaceae* | *Spirosoma* |
| Linden_Middle | ASV78 | *Hymenobacteraceae* | *Hymenobacter* |

| **Host** | **ASV** | **Family** | **Genus** |
| --- | --- | --- | --- |
| Linden_Bottom | ASV70 | *Erwiniaceae* | *Pantoea* |
| Linden_Bottom | ASV47 | *Nocardiaceae* | *Rhodococcus* |
| Linden_Bottom | ASV100 | *Oxalobacteraceae* | *Massilia* |
| Linden_Bottom | ASV89 | *Microbacteriaceae* | *Rathayibacter* |
| Linden_Bottom | ASV83 | *Sphingobacteriaceae* | *Pedobacter* |
| Linden_Bottom | ASV75 | *Spirosomaceae* | *Spirosoma* |

b)

| **Host** | **ASV** | **Family** | **Genus** |
| --- | --- | --- | --- |
| Oak_Top | ASV179 | *Beijerinckiaceae* | *1174-901-12* |
| Oak_Top | ASV125 | *Acetobacteraceae* | *Acidiphilium* |
| Oak_Top | ASV69 | *Pseudomonadaceae* | *Pseudomonas* |
| Oak_Top | ASV145 | *Pseudomonadaceae* | *Pseudomonas* |
| Oak_Top | ASV92 | *Acetobacteraceae* | |
| Oak_Top | ASV141 | *Spirosomaceae* | *Spirosoma* |
| Oak_Middle | ASV27 | *Erwiniaceae* | *Erwinia* |
| Oak_Middle | ASV145 | *Pseudomonadaceae* | *Pseudomonas* |
| Oak_Bottom | ASV151 | *Pseudomonadaceae* | *Pseudomonas* |
| Oak_Bottom | ASV55 | *Erwiniaceae* | *Erwinia* |
| Oak_Bottom | ASV135 | *Oxalobacteraceae* | *Massilia* |
| Oak_Bottom | ASV182 | *Spirosomaceae* | *Spirosoma* |
| Oak_Bottom | ASV41 | *Pseudomonadaceae* | *Pseudomonas* |
| Ash_Top | ASV102 | *Acetobacteraceae* | *Acidiphilium* |
| Ash_Top | ASV178 | *Spirosomaceae* | *Spirosoma* |
| Ash_Top | ASV99 | *Beijerinckiaceae* | *Methylocella* |
| Ash_Top | ASV179 | *Beijerinckiaceae* | *1174-901-12* |
| Ash_Middle | ASV164 | *Myxococcaceae* | *P3OB-42* |

| **Host** | **ASV** | **Family** | **Genus** |
| --- | --- | --- | --- |
| Ash_Middle | ASV150 | *Spirosomaceae* | *Huanghella* |
| Ash_Middle | ASV137 | *Hymenobacteraceae* | *Hymenobacter* |
| Ash_Middle | ASV176 | *Acetobacteraceae* | *Acidiphilium* |
| Ash_Middle | ASV7 | *Oxalobacteraceae* | *Massilia* |
| Ash_Bottom | ASV131 | *Sphingomonadaceae* | *Sphingomonas* |
| Ash_Bottom | ASV178 | *Spirosomaceae* | *Spirosoma* |
| Linden_Top | ASV134 | *Morganellaceae* | *Incertae Sedis* |
| Linden_Top | ASV193 | *Sphingomonadaceae* | *Sphingomonas* |
| Linden_Top | ASV55 | *Erwiniaceae* | *Erwinia* |
| Linden_Top | ASV141 | *Spirosomaceae* | *Spirosoma* |
| Linden_Top | ASV83 | *Sphingobacteriaceae* | *Pedobacter* |
| Linden_Middle | ASV134 | *Morganellaceae* | *Incertae Sedis* |
| Linden_Middle | ASV83 | *Sphingobacteriaceae* | *Pedobacter* |
| Linden_Bottom | ASV182 | *Spirosomaceae* | *Spirosoma* |
| Linden_Bottom | ASV121 | *Erwiniaceae* | *Izhakiella* |
| Linden_Bottom | ASV125 | *Acetobacteraceae* | *Acidiphilium* |
| Linden_Bottom | ASV10 | *Pseudomonadaceae* | *Pseudomonas* |
| Linden_Bottom | ASV134 | *Morganellaceae* | *Incertae Sedis* |
| Linden_Bottom | ASV102 | *Acetobacteraceae* | *Acidiphilium* |
| Linden_Bottom | ASV189 | *Spirosomaceae* | *Spirosoma* |
